# Mechanisms and therapeutic strategies of asthma: from bench to bedside

**DOI:** 10.1038/s41392-026-02853-8

**Published:** 2026-08-10

**Authors:** Cong Xie, Ying Wei, Chang Su, Maimaititusun Yalikun, Yiwei Chu, Hang Yu, Meiling Jin, Weifang Xu, Jiangtao Lin, Jingcheng Dong

**Affiliations:** 1 Department of Integrative Medicine, Huashan Hospital Affiliated to Fudan University, Fudan Institutes of Integrative Medicine, Fudan University Shanghai Medical College, Shanghai, PR China; 2 Yuquan Hospital, School of Clinical Medicine, Tsinghua Medicine, Tsinghua University, Beijing, PR China; 3 Department of Biomedical Sciences, City University of Hong Kong, Hong Kong, PR China; 4 MOE Innovative Center for New Drug Development of Immune Inflammatory Diseases, Faculty of Immunology, School of Basic Medical Sciences, Fudan University, Shanghai, PR China; 5 Charité - Universitätsmedizin Berlin, corporate member of Freie Universität Berlin and Humboldt-Universität zu Berlin, Berlin, Germany; 6 Department of Allergy, Zhongshan Hospital Affiliated to Fudan University, Shanghai, PR China; 7 Department of Pulmonary and Critical Care Medicine, Shenzhen Hospital (Futian) of Guangzhou University of Chinese Medicine, Shenzhen, PR China; 8 National Center for Respiratory Medicine, National Clinical Research Center for Respiratory Diseases, Department of Pulmonary and Critical Care Medicine, Center of Respiratory Medicine, China-Japan Friendship Hospital, Beijing, PR China

**Keywords:** Respiratory tract diseases, Inflammation, Biomarkers

## Abstract

Asthma is a heterogeneous chronic airway disease arising from a complex interplay of genetic susceptibility and environmental exposures. Key pathobiological features include dysregulated immune responses (particularly type 2 inflammation), structural airway remodeling, and mucus hypersecretion. Recent advances have illuminated mechanisms from epithelial “alarmin” cytokine release (TSLP, IL-33, IL-25) to downstream cellular networks involving Th2/Th17 lymphocytes, group 2 innate lymphoid cells (ILC2s), eosinophils, and mast cells. These pathways converge on bronchial hyperresponsiveness and airflow obstruction. Traditional therapy with inhaled corticosteroids and bronchodilators has improved asthma control, yet many patients, especially those with non–type 2-driven endotypes such as neutrophilic or obesity-related asthma, remain suboptimally controlled. This limitation has driven the development of precision medicine approaches that target specific cytokines and signaling cascades, including IgE, IL-5, IL-4/13, and TSLP, as well as intracellular signaling processes such as JAK–STAT. Parallel innovations in biomarkers such as FeNO, blood eosinophils, periostin, and multi-omics-based signatures facilitate patient stratification and prediction of treatment response. This comprehensive review synthesizes current knowledge from genetic and epigenetic foundations of asthma, through immunologic and neurogenic mechanisms, to translational advances in therapeutics from laboratory to clinic. We highlight the importance of endotyping and “treatable traits” such as eosinophilia, mucus plugging, or small-airway dysfunction in guiding individualized therapy. The emerging paradigm aims not only for symptom control but also for disease modification and remission, leveraging biomarkers and multidisciplinary approaches to achieve long-term asthma control in a broad patient population.

## Introduction

Asthma is one of the most common chronic diseases globally, affecting an estimated 300 million people with rising prevalence in many regions.^1,2^ Despite being preventable and treatable, asthma continues to cause significant morbidity and mortality. Approximately 1000 people die from asthma every day worldwide, many of them young, and most of these deaths could be avoided.^3^ The economic burden is also substantial, including direct medical costs (hospitalizations, medications) and indirect costs from lost productivity. Asthma’s impact is particularly high in low- and middle-income countries where underdiagnosis and undertreatment are prevalent, contributing to excess emergency visits and deaths.^4^

Standard therapy for persistent asthma has long centered on inhaled corticosteroids (ICS) to suppress airway inflammation, often combined with inhaled long-acting β_2_-agonists (LABA) for bronchodilation.^5^ Additional controllers, such as leukotriene receptor antagonists (LTRAs) and theophylline, are used in select cases. These treatments have improved symptom control and reduced exacerbations for many patients. However, limitations remain. A significant subset of patients with asthma has uncontrolled disease despite high-dose ICS/LABA, or experiences frequent exacerbations upon tapering oral corticosteroids.^6^ The side effects of chronic corticosteroid use (e.g., adrenal suppression, osteoporosis) pose clinical challenges,^7^ and medication adherence is often suboptimal. Moreover, conventional therapies largely target T2-high (type 2 inflammation) eosinophilic asthma; patients with non-T2 inflammation (e.g., neutrophilic or paucigranulocytic asthma) often respond poorly to ICS, reflecting an unmet need for alternative strategies.^8^ Difficult-to-treat populations include obese asthmatics, smokers, and those with predominant small airway disease, phenotypes in which inflammation and remodeling may not be driven by classical allergic Th2 pathways.^9^

Asthma management paradigms have evolved significantly in recent years. A major shift has been the move away from sole reliance on short-acting β_2_-agonists (SABA) as quick-relief therapy.^10^ It is now recognized that regular or overuse of SABAs without anti-inflammatory treatment can paradoxically worsen control and increase the risk of exacerbations.^11^ Current international guidelines recommend the early introduction of ICS even in mild asthma, often via an “anti-inflammatory reliever (AIR)” approach using a combination of low-dose ICS-formoterol as needed instead of SABA alone.^12^ The ICS-formoterol single maintenance and reliever therapy (SMART) strategy has shown superior prevention of exacerbations compared to traditional regimens, heralding a new era of proactive anti-inflammatory treatment for even mild asthma.^13^

Despite these advances, considerable gaps persist. Patients with severe asthma (estimated ~5–10% of asthmatics) account for a disproportionate share of disease burden, as they suffer from frequent symptoms and life-threatening exacerbations despite maximal standard therapy.^14,15^ Within this group, heterogeneity is now appreciated. Some have classic allergen-driven eosinophilic inflammation, while others have nonatopic asthma with airway neutrophilia, often associated with environmental tobacco smoke exposure or pollution.^16^ Some asthmatics experience persistent airflow limitation from fixed airway remodeling, and others have excess mucus plugging in small bronchi that contributes to airflow obstruction and acute attacks. Comorbidities such as chronic rhinosinusitis with nasal polyps (CRSwNP), gastroesophageal reflux disease, and anxiety can further complicate management. These varied factors underscore the need for a more personalized, mechanism-based approach to asthma care.

Over the past two decades, there has been an explosion of research from bench to bedside uncovering the cellular and molecular underpinnings of asthma. Key discoveries include the role of airway epithelium-derived cytokines (i.e., “alarmins”) in orchestrating type 2 immunity,^17^ the identification of genetic loci conferring asthma susceptibility, and the delineation of distinct inflammatory endotypes (T2-high vs T2-low). These insights have directly led to targeted therapies, most notably, monoclonal antibody biologics that neutralize IgE or specific interleukins, which have transformed outcomes for many patients with severe asthma.^18^ The anti-IgE antibody omalizumab was the first successful example of immune-targeted asthma treatment, significantly reducing exacerbations in allergic asthmatics.^19^ Subsequently, inhibitors of IL-5 and its receptor ushered in effective therapy for eosinophilic asthma, and more recently, an antibody against IL-4 receptor α (blocking both IL-4 and IL-13 signaling) has shown broad efficacy in T2-high asthma, including in atopic comorbidities.^20^ The FDA approval of tezepelumab (anti-TSLP) marked an important milestone, as it targets an upstream epithelial cytokine and has demonstrated a reduction in exacerbations even in patients with low eosinophil counts.^21^ Meanwhile, for T2-low asthma, strategies such as long-term macrolide antibiotics have shown benefits in reducing exacerbations irrespective of eosinophilic status,^22^ although optimal treatments for these phenotypes remain an area of active investigation.

Beyond biologics, emerging therapies in the pipeline include inhaled small-molecule drugs aimed at intracellular signaling (e.g., JAK-STAT inhibitors, kinase modulators) and novel modalities such as gene editing and cell-based therapies. Precision medicine is further enabled by improved biomarkers (such as exhaled nitric oxide, blood eosinophils, periostin and even breath volatile organic compounds) that can guide therapy selection and monitor response. Researchers are also developing integrative “omics” approaches and machine-learning models to parse asthma complexity and predict individual treatment responses.^23^

This comprehensive review aims to systematically summarize the current understanding of asthma pathobiology and the translational progress in therapy, with an emphasis on linking mechanisms to clinical targets. We will discuss the genetic, epigenetic and environmental foundations of asthma, dissect the pathophysiological and immunological mechanisms and review the full spectrum of therapeutic strategies from conventional drugs to cutting-edge precision biologics and beyond. We also consider future perspectives, including the integration of multidimensional data and ensuring the global accessibility of advanced treatments. Through this bench-to-bedside lens, we highlight how asthma care is moving toward phenotype-driven and personalized interventions, and we outline the challenges that remain in achieving optimal outcomes and eventual disease remission for all patients.

## Genetic, epigenetic, and environmental regulation

Asthma risk is shaped by a combination of inherited genetic factors, epigenetic modifications, and diverse environmental exposures. These factors interact to determine immune development and airway responsiveness early in life, ultimately producing the clinical asthma phenotype in susceptible individuals.

### Genetic susceptibility and GWAS findings

Family and twin studies indicate that asthma has a significant heritable component, estimated at ~35–70% liability.^24^ Unlike monogenic disorders, asthma’s inheritance is polygenic and involves many loci, each conferring modest risk. Early linkage studies and candidate-gene approaches implicated immune genes (e.g., *IL4*, *IL13*, *ADAM33*), but the advent of large-scale genome-wide association studies (GWAS) revolutionized our understanding.^25^ Over the past 15 years, GWAS have identified ~150–200 loci associated with asthma susceptibility. Each locus typically has an odds ratio of ~1.1–1.5 per risk allele, underscoring that no single gene “causes” asthma; rather, it is the aggregate burden of many variants that contribute to risk, reflected in emerging polygenic risk scores (PRS).^26^ Notably, known variants still account for only a minority of the estimated heritability, the so-called “missing heritability,”^27^ pointing to additional undiscovered or gene–environment components.

One of the most consistently replicated asthma loci is on chromosome 17q12**–**21,^28^ a region containing the *ORMDL3* and *GSDMB* genes. This locus was the top hit in the first asthma GWAS and is primarily associated with childhood-onset asthma.^29^ *ORMDL3* and *GSDMB* are not classical immune genes; *ORMDL3* regulates sphingolipid metabolism and endoplasmic reticulum stress responses, while *GSDMB* (gasdermin B) is involved in epithelial cell pyroptosis.^30^ Their association with asthma suggests that fundamental cellular processes (e.g., apoptosis, barrier integrity) are important in disease susceptibility. Another top locus is on chromosome 5q, encompassing the *IL-33* and *IL1RL1* genes. IL-33 is an epithelial-derived alarmin strongly linked to allergic inflammation, and *IL1RL1* encodes its receptor ST2 expressed on many immune cells. Variants near *IL1RL1* were identified in multiancestry GWAS. Similarly, the *TSLP* gene (thymic stromal lymphopoietin, another epithelial cytokine) on 5q22 has risk variants associated with asthma and allergic disorders. Genes coding for cytokine receptors, such as *IL4R* (the IL-4/IL-13 receptor α chain) and *IL5RA*, have functional polymorphisms influencing signaling and asthma risk; examples include the IL4R Q576R variant, which affects responsiveness to IL-4/13.^31^ Other notable loci include *RAD50/IL13* on 5q31 (involved in IL-13 regulation),^32^ *HLA-DQ* and other MHC region genes (especially linked to childhood onset and atopy), *ADAM33* (implicated in airway remodeling),^33^ and *CHRD* (chr11, possibly related to TGF-β signaling).

Initial GWAS were heavily European-centric. Recent multiancestry meta-analyses, involving tens of thousands of cases across diverse populations, have expanded the catalog of risk variants. The Trans-National Asthma Genetic Consortium (TAGC) meta-analysis in 2018 identified 17 new loci among 67 total hits, including the *GATA3* locus,^34^ which had been underpowered in individual GWAS before. A subsequent meta-analysis by Johansson et al. (2019) in >350,000 individuals reported 141 asthma-associated loci (41 novel) shared with hay fever and eczema, reflecting the genetic overlap among allergic diseases.^35^ More recently, a GWAS by Olafsdottir et al. (2020) combining the UK Biobank and other cohorts uncovered 88 independent risk variants at 56 loci, including novel low-frequency protective variants in genes such as *TNFRSF8* (CD30) and *TGFBR1*, pointing to roles for TNF receptor signaling and TGF-β pathways in asthma susceptibility.^36^ Intriguingly, that study also highlighted a rare loss-of-function variant in *IL33* that protects against asthma,^37^ bolstering evidence that IL-33 is a key upstream driver and validating it as a therapeutic target in asthma. The rich GWAS data now enable calculation of PRS, which aggregates an individual’s risk alleles into a single score.^38,39^ PRS can modestly predict asthma development and may inform, in the future, early-life preventive strategies when combined with environmental risk assessment.^40,41^ Notably, PRS performance improves in non-European populations when multiancestry data are included, underscoring the importance of diversity in genomic studies.^42^

A major challenge is moving from GWAS hits to causal genes and biological insights. Many loci lie in noncoding regions, affecting gene expression (i.e., expression quantitative trait loci, eQTLs)^43^ rather than protein structure. Fine-mapping and functional genomics are identifying which genes are truly driving the associations.^44^ For instance, at 17q21, the risk variants increase *ORMDL3/GSDMB* expression in airway cells,^45^ and experiments confirm ORMDL3’s role in calcium homeostasis and inflammatory cascades.^46^ At the *IL1RL1* locus, genetic variants were found to correlate with IL1RL1 (ST2) protein levels and DNA methylation status, providing a link between genotype and the inflammatory milieu.^47^ Another example is GSDMB, whose protein is involved in pyroptosis; a recent study suggested that a genetic variant creates alternative splicing that could alter epithelial cell death pathways in asthmatic airways.^48^ These mechanistic insights not only validate the GWAS approach but also point to novel therapeutic targets. Indeed, pathways highlighted by genetics (TSLP, IL-33/ST2, IgE regulation via IL-4/IL-13, etc.) correspond to many of the new targeted therapies being developed or already in use in asthma.^49^

### Epigenetic modifications and transcriptomic regulation

Epigenetics, defined as heritable changes in gene expression that are not due to DNA sequence changes, offers a mechanistic link between environment and gene function in asthma. Airway inflammation and remodeling in asthma are accompanied by widespread epigenetic modifications in immune and epithelial cells.^50^ Key epigenetic marks include DNA methylation of cytosine–guanine (CpG) dinucleotides, post-translational modifications of histone proteins such as acetylation and methylation, and noncoding RNAs including microRNAs. These changes can persist or be “imprinted” by environmental exposures, potentially explaining phenomena such as the developmental origins of asthma.^51^

Epigenome-wide association studies (EWAS) have cataloged differences in DNA methylation between asthmatics and non-asthmatics.^52^ For example, one EWAS identified differential methylation at genes related to the epithelial barrier and immunity (such as *ADAMTSL3* and *IL2RB*) in children who develop asthma. Another study found that asthmatic children had distinct methylation signatures in T cells, including at the *IL4* and *IFNG* gene loci, correlating with skewed Th2/Th1 balance.^53^ Interestingly, some methylation changes overlap with genetic risk regions from GWAS.^54^ Intersection of GWAS and EWAS data has highlighted certain genes, *SMAD3*, *GATA3*, *IL5*, and *ORMDL3*, that show both risk SNPs and differential methylation in asthma,^55^ suggesting these are crucial nodes in the gene–environment network of asthma pathogenesis.^56^ Environmentally induced epigenetic changes can modulate gene expression; for instance, tobacco smoke exposure in utero and in early life is associated with increased DNA methylation of the AHRR gene in blood DNA, which correlates with higher asthma risk and reduced lung function in children.^57,58^ Air pollution exposure has been linked to methylation changes in the *FOXP3* gene (critical for regulatory T cells [Tregs]) and the *ACSL3* gene in airway cells, potentially diminishing immune regulation and enhancing allergic inflammation.^59,60^

Asthmatic inflammation has also been tied to changes in histone acetylation and methylation, which govern chromatin accessibility.^61^ In general, histone acetylation by histone acetyltransferases (HATs) promotes gene transcription, while deacetylation by histone deacetylases (HDACs) inhibits it.^62^ In asthma, increased acetylation at promoters of Th2 cytokine genes (e.g., IL5, IL13) has been observed, facilitating their expression. Conversely, some anti-inflammatory genes may be silenced by repressive histone marks.^63^ One well-known finding is that HDAC2 expression/activity is reduced in peripheral blood and alveolar macrophages of severe asthmatics, especially those with corticosteroid resistance.^64^ HDAC2 loss leads to the accumulation of acetylated histones on inflammatory gene promoters (IL-8, TNFα, etc.), driving persistent inflammation and conferring steroid insensitivity since corticosteroids require HDAC2 to repress gene transcription.^65^ Oxidative stress from smoking or severe inflammation can inactivate HDAC2, providing one mechanism for steroid resistance.^66^ Encouragingly, HDAC2-targeting therapies are being explored: theophylline at low doses was shown to restore HDAC activity and improve steroid sensitivity in vitro,^67^ and HDAC-boosting strategies or inhibitors of HATs might help restore balance. Other histone marks, such as methylation of H3K4 (activating) or H3K27 (repressive), have been implicated; for example, pediatric studies found that persistent wheezing and later asthma were associated with differences in H3K27 methylation at certain loci in T cells.^68^

Noncoding RNAs add another regulatory layer. MicroRNAs (miRNAs) are ~22-nucleotide RNAs that typically suppress target mRNA translation.^69^ Numerous miRNAs are dysregulated in asthma and contribute to either exacerbating or dampening inflammation. For example, miR-155 and miR-21 are often upregulated in asthma and are known to amplify type 2 inflammation.^70^ miR-155 can enhance Th2 cell activity, and miR-21 suppresses negative regulators of IL-13 signaling. In mouse models, knocking out miR-21 reduced eosinophilic inflammation, highlighting its role. On the other hand, some miRNAs (e.g., let-7 family, miR-146a) act as anti-inflammatory brakes; reduced levels of these have been noted in asthma. Long noncoding RNAs (lncRNAs) are >200 nt transcripts that can regulate gene expression via various mechanisms.^71^ Asthma transcriptomic studies have identified lncRNAs that are differentially expressed; for example, the lncRNA GATA3-AS1 regulates the Th2 master transcription factor GATA3, potentially influencing Th2 differentiation.^72^ Although lncRNA research in asthma is still emerging, these molecules could serve as both biomarkers and therapeutic targets.

Epigenetic modifications also underlie changes in the airway structure and “hardwiring” of chronic asthma.^73^ Airway smooth muscle cells from asthmatics show a different epigenetic landscape that perpetuates hypercontractility and excess proliferation, a concept termed “epigenetic memory” of the asthmatic airway. For example, one study demonstrated that asthmatic smooth muscle cells maintained elevated production of extracellular matrix proteins even after weeks in culture, linked to stable changes in histone marks at profibrotic gene loci.^74^ Targeting these epigenetic marks with inhibitors of bromodomain proteins that bind to acetylated histones could “reset” cells toward a non-asthmatic phenotype.^75,76^ Early preclinical studies using HDAC or DNA methylation inhibitors demonstrated their ability to modulate airway inflammation.^77^ In animal models, a DNA methyltransferase inhibitor decreased IL-13 levels and reduced airway hyperresponsiveness, suggesting novel therapeutic possibilities.^77,78^

Crucially, epigenetic marks often mediate gene–environment interactions.^79^ A child’s environmental exposures, such as farm living, air pollution, and nutrition, can leave epigenetic “footprints” that alter gene expression without changing the genetic code.^80^ The “hygiene hypothesis” and its extension, the “epithelial barrier hypothesis,”^81^ posit that changes in Western lifestyles, including reduced microbial contact and increased exposure to pollutants and chemicals, lead to epigenetic alterations in epithelial and immune cells that disrupt immune tolerance and raise the risk of asthma.^82^ For example, children who grow up on traditional farms with abundant microbial dust exposure exhibit epigenetic differences in pattern-recognition receptor genes and Th1/Th2 balance genes, potentially explaining their lower asthma rates.^80^ One investigation reported that the protective effect of a farm childhood on asthma was far stronger among individuals carrying a risk allele in *TNFAIP3*, an immune regulatory gene, illustrating how genetic susceptibility can be modified by environmental exposure through epigenetic and related mechanisms.^83,84^

The reversibility of epigenetic modifications offers an intriguing therapeutic opportunity.^85^ Agents such as HDAC inhibitors, already in clinical use for certain cancers, and small molecules that modulate specific miRNAs are being explored for their potential in inflammatory disorders.^63^ In asthma, miRNA-based interventions may employ mimics to restore protective miRNAs or antagomirs to silence those that drive inflammation.^86^ One candidate currently under investigation is a miR-155 inhibitor designed to attenuate allergic inflammation. Another promising strategy involves inhaled small interfering RNAs (siRNAs) directed against key molecular drivers such as IL4Rα or TSLP to locally suppress pathogenic pathways.^87^ Although none of these approaches have yet reached clinical practice for asthma, early trials, for example, the successful delivery of an inhaled siRNA against respiratory syncytial virus, demonstrate the feasibility of pulmonary administration.^88^ Current research is mapping the asthma epigenome to identify biomarkers such as DNA methylation signatures that predict exacerbation risk and to develop therapies capable of reprogramming immune cells toward tolerance. Ultimately, integrating epigenetic biomarkers with clinical data may enable clinicians to identify patients with steroid-resistant inflammation driven by an “epigenetic lock” and to tailor adjunctive treatments that specifically target those molecular pathways.

### Microbiome and exposome

**Fig. 1:**
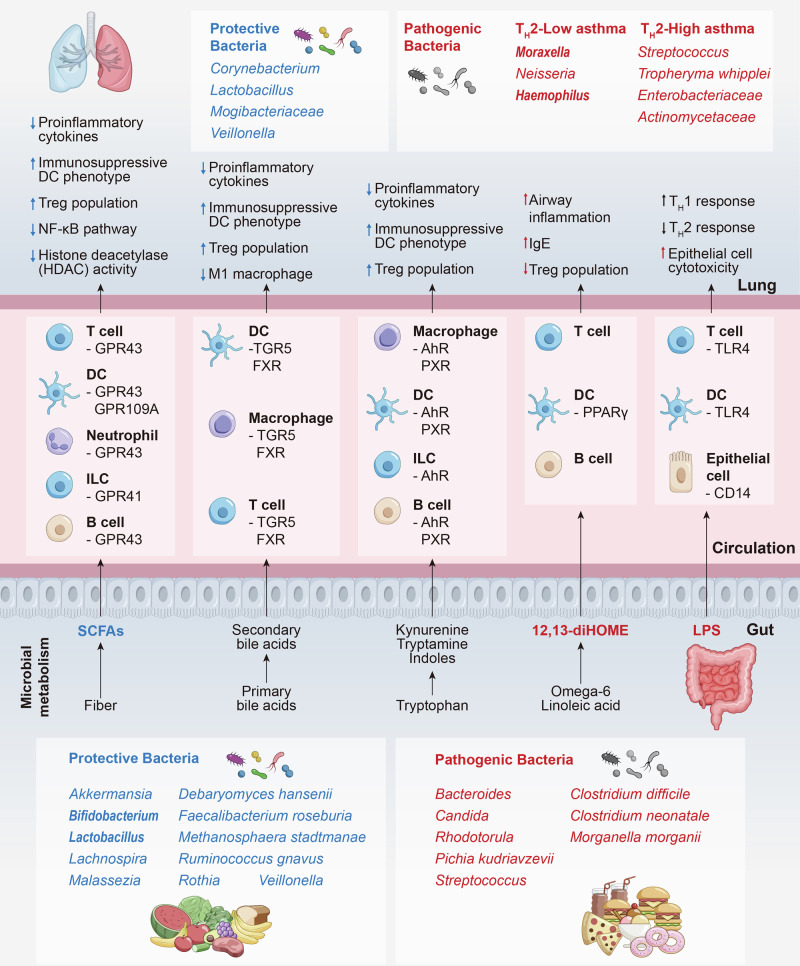
The gut–airway microbiome axis in asthma. The gut–lung axis concept suggests that microbial populations in the gastrointestinal tract can influence systemic immune responses, including in the airways. Likewise, the airway microbiome itself (the bacteria, viruses, and fungi residing in the respiratory tract) may differ in asthmatics vs. healthy people and contribute to chronic inflammation. GPR G protein-coupled receptor, TGR5 G protein-coupled bile acid receptor Gpbar1, FXR farnesoid X receptor, AhR aryl hydrocarbon receptor, PXR pregnane X receptor, PPAR peroxisome proliferator activated receptor, TLR toll-like receptor, SCFA short-chain fatty acid, 1213-diHOME 1213-dihydroxy-9Z-octadecenoic acid, LPS lipopolysaccharides. This figure was crafted entirely by hand (no AI tools) by the author(s) using Adobe Illustrator

The respiratory tract is constantly exposed to a myriad of microbial and environmental stimuli, collectively known as the exposome.^89^ Recent research has established that asthma pathogenesis is strongly shaped by the microbiome–immune–lung axis, through which microbial communities in the airways and gut interact with the host immune system to influence respiratory health.^90^ Imbalances in these microbial populations, known as “dysbiosis,” are associated with abnormal immune responses and the onset of asthma (Fig. 1).^91^

During early life, the **gut microbiome** plays a critical role in training the immune system.^92^ Infants who later develop asthma often exhibit distinct gut bacterial profiles in the first months of life, with reduced levels of bacteria that produce immunoregulatory short-chain fatty acids (SCFAs).^93^ Lower colonization by *Lactobacillus* and *Bifidobacterium*, as well as delayed microbial diversification,^94^ have been linked to an increased risk of asthma. These beneficial bacteria ferment dietary fibers into metabolites such as butyrate and propionate,^95^ which promote Treg development and strengthen the gut–lung immune connection. A landmark study demonstrated that the loss of specific gut commensals in infants leads to reduced production of key microbial metabolites that normally support airway immune tolerance.^96^ Supplementation of these metabolites in germ-free mice prevented experimental asthma, revealing a causal chain from gut microbes to lung immunity.^96^

The **airway microbiome** itself is also altered in asthma.^97^ In healthy individuals, the lungs harbor a low-biomass yet diverse microbial community resembling that of the oral cavity.^98,99^ In contrast, asthmatic airways, particularly those affected by moderate-to-severe disease, are frequently colonized by bacteria from the *Proteobacteria* phylum, including *Haemophilus*, *Moraxella*, and *Klebsiella* species, while exhibiting decreased abundance of *Bacteroidetes* and *Firmicutes*.^91^ These compositional changes may both result from and contribute to airway inflammation. For example, the presence of *Haemophilus influenzae* in sputum is associated with neutrophilic asthma and can induce IL-8 and other pro-neutrophil signals.^100^ The causality is likely bidirectional: persistent inflammation may create a niche that supports certain microbial populations, which in turn sustain or amplify the inflammatory response. Clinical evidence further suggests that microbiome modulation may influence disease outcomes. Macrolide antibiotic therapy has been shown to induce modest but measurable shifts in the airway microbiome, and these changes correlate with fewer asthma exacerbations.^22^ This finding implies that part of the therapeutic benefit of macrolides may stem from their capacity to reshape microbial communities and dampen inflammation.^101^

**Fig. 2:**
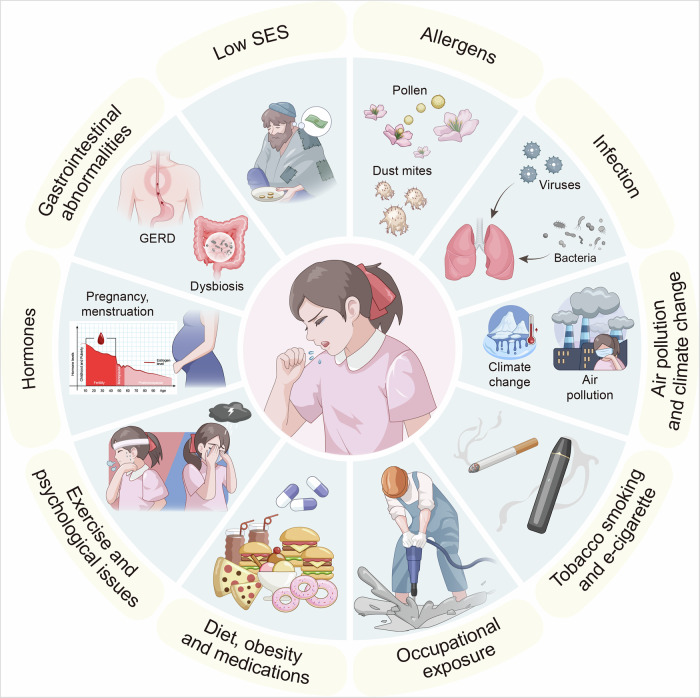
Environmental and exposomal factors contributing to asthma pathogenesis. **A**llergens—house dust mite, pollen, molds, animal dander, cockroach, and fungal spores. **B**ronchial infections—rhinovirus, respiratory syncytial virus, influenza, and atypical bacteria. **C**limate and weather extremes—cold air, wildfire smoke, dust storms, and thunderstorms. **D**rugs—aspirin, nonsteroidal anti-inflammatory drugs (NSAIDs), and β-blockers. **E**xercise and emotions—physical exertion, hyperventilation, laughter, anxiety, low socioeconomic status (SES), and acute psychosocial stress. **F**ood-dietary allergens (milk, egg, peanut, shellfish), food additives such as sulfites or benzoates, and nutritional imbalance related to obesity or micronutrient deficiency. **G**astroesophageal reflux disease (GERD)—acid reflux, microaspiration, and nocturnal cough aggravating airway hyperreactivity. **H**ormones—sex hormone fluctuations during puberty, menstruation, pregnancy, or menopause influencing asthma control and severity. **I**rritants—tobacco smoke (active and passive), e-cigarette aerosols, perfume, indoor pollutants, industrial chemicals, and volatile organic compounds. **J**ob—isocyanates, cleaning agents, flour dust, wood dust, and animal proteins. This figure was hand-rendered by the author(s) using Adobe Illustrator, not by an AI algorithm

**Environmental exposures** play a central role in shaping both the microbiome and immune responses, influencing asthma susceptibility and progression. Major exposures include allergens, air pollution, viral infections, tobacco smoke, diet and obesity, occupational and indoor irritants, as well as climate-related factors (Fig. 2).

#### Allergens

High levels of house dust mites, cockroaches, or fungal spores can drive allergic sensitization in genetically predisposed individuals.^102^ Repeated allergen contact in the lower airways induces a Th2-dominant mucosal immune response. In contrast, environments with diverse microbial and allergenic exposures, such as farms, can foster immune tolerance.^103^ Children raised in rural farming settings have a markedly lower risk of asthma and atopy, partly due to inhalation of microbial components such as lipopolysaccharide (LPS), which activates innate immune receptors such as TLR4 and promotes Th1 and Treg responses over Th2 polarization.^104^ These children also exhibit stronger antiviral gene expression and reduced IgE sensitization.^104^

#### Air pollution

Exposure to traffic-related pollutants and fine particulate matter (PM_2.5_, PM_10_) is a well-established driver of asthma onset and exacerbation.^105^ Pollutants such as diesel exhaust particles damage the airway epithelium, induce oxidative stress, and activate NF-κB and related inflammatory pathways.^106^ They also skew dendritic cell function toward allergic sensitization and can alter the airway microbiome by favoring resilient, potentially pathogenic species.^107,108^ In addition, pollution can cause epigenetic modifications, including DNA methylation changes in inflammatory genes, which may have lasting effects on immune regulation.^109^

#### Viral infections

Respiratory viruses, particularly rhinovirus (RV) and respiratory syncytial virus (RSV), are major triggers of asthma development and exacerbation.^110,111^ Severe RSV bronchiolitis in infancy is strongly associated with persistent wheezing and later asthma.^111^ These viruses disrupt epithelial integrity and immune homeostasis, while RV infection promotes epithelial release of IL-25, IL-33, and TSLP—alarmins that amplify type 2 inflammation in atopic individuals.^112^ Viral infections can also reshape airway bacterial communities, predisposing them to secondary bacterial overgrowth and sustained inflammation.^113,114^

#### Tobacco smoke

Maternal smoking during pregnancy and exposure to secondhand smoke in early childhood substantially increase the risk of developing asthma.^115^ Prenatal smoke exposure impairs fetal lung growth, resulting in reduced lung function at birth and induces lasting alterations in immune development. These effects are partly mediated by epigenetic modifications in the offspring’s immune cells.^116^ For example, hypermethylation of the *RUNX3* gene—a key transcription factor regulating Th1 and Th2 differentiation—has been detected in the cord blood of infants born to mothers who smoked, suggesting a predisposition toward Th2-dominant immune responses.^117^ In children with asthma, exposure to secondhand smoke is a well-known aggravating factor that worsens symptoms and reduces responsiveness to corticosteroids.^118^ Experimental studies indicate that smoke-induced oxidative stress activates the phosphoinositide-3-kinase (PI3K) signaling pathway in airway epithelial cells, leading to decreased HDAC2 activity and consequent steroid resistance.^66^ Eliminating both active and passive tobacco smoke exposure remains an essential strategy in preventing asthma onset and improving disease control.

#### Diet and obesity

Diet profoundly influences the gut microbiome and systemic immune tone. High-fiber diets rich in fruits, vegetables, and whole grains are linked to reduced asthma risk,^119^ largely through increased microbial production of SCFAs with anti-inflammatory effects. Conversely, Western diets high in refined sugars and fats promote proinflammatory microbial profiles.^120^ Obesity adds a metabolic and inflammatory dimension, as adipose tissue secretes adipokines such as leptin and adiponectin that modulate airway inflammation.^121^ Obese individuals often display a distinct asthma phenotype characterized by neutrophilic inflammation and altered metabolic signaling. Low levels of adiponectin may enhance type 2 airway inflammation by diminishing AMPK-mediated feedback inhibition of IL-33/NF-κB signaling in immune cells such as ILC2s, thereby linking metabolic dysregulation to asthma pathogenesis.^122^

#### Occupational and indoor exposures

Long-term exposure to workplace sensitizers, including flour dust, isocyanates, wood dust, and animal dander, can lead to occupational asthma.^123^ Chronic exposure to irritants such as cleaning agents or ozone can trigger nonallergic asthma through neural and innate immune mechanisms. Indoor factors such as mold, dampness, and endotoxin levels may either exacerbate symptoms or, under specific conditions and timing, confer protection, as observed in farm environments.^124^

#### Climate change and extreme weather events

Climate-related exposures are emerging as important asthma determinants. Wildfire smoke, rich in fine particulate matter and toxic chemicals, has been associated with spikes in emergency visits for asthma.^125^ Thunderstorm asthma—triggered when storms fragment pollen grains into respirable particles—can cause epidemic surges of acute asthma attacks, as seen in Melbourne in 2016.^126^ Rising atmospheric CO_2_ and global temperatures are lengthening pollen seasons and increasing pollen allergenicity, trends expected to worsen allergic asthma worldwide.^127^ Public health measures such as pollen forecasting, air quality alerts, and environmental controls are increasingly essential components of asthma management.

The microbiome and exposome together define the ecological and immunological landscape in which asthma develops. Maintaining a balanced microbiome through a healthy diet and possibly probiotic or prebiotic supplementation while minimizing harmful exposures such as pollutants, tobacco smoke, and occupational irritants represents a comprehensive approach to asthma prevention. Emerging interventions, including bacterial lysates and synbiotics, aim to strengthen immune tolerance early in life, shifting focus from managing downstream inflammation to modulating upstream environmental and microbial influences. The multifactorial nature of these interactions underscores that asthma is as much an environmental and microbial disease as it is a genetic disease, requiring integrated management that combines environmental control with pharmacologic therapy.

### Gene–environment interactions

Asthma’s remarkable clinical heterogeneity reflects the complex interplay between genetic predisposition and environmental influences.^128^ No single determinant can account for its onset; rather, asthma emerges when multiple genetic vulnerabilities coincide with permissive environmental exposures. Understanding these gene–environment interactions is central to identifying at-risk individuals and developing effective prevention strategies.

A clear example of such interaction is between viral infection and a genetic risk locus on 17q21.^129^ Children carrying 17q21 risk variants (*ORMDL3*/*GSDMB*) have a substantially higher likelihood of developing wheezing illnesses and asthma following RV infection in infancy.^130^ The variant appears to weaken antiviral defenses or intensify virus-induced airway inflammation by promoting epithelial release of cytokines such as IL-25 and IL-33.^131^ Longitudinal birth cohort studies confirm that children with these risk alleles who experienced RV-associated wheezing early in life had significantly higher asthma prevalence by school age than noncarriers.^130^

Another notable interaction occurs between air pollution and antioxidant genes.^132^ Variants in *GSTM1*/*GSTP1*, *NQO1*, and *catalase* that reduce antioxidant capacity magnify the inflammatory effects of pollutants such as ozone and diesel exhaust.^133,134^ Children with these polymorphisms exposed to high pollution show greater airway inflammation, impaired lung growth, and higher asthma incidence compared with those carrying more protective alleles.^135^ In this context, environmental oxidative stress acts synergistically with genetic vulnerability to promote disease.

Diet and microbial exposures also modify asthma risk through gene–environment coupling. Variants in *CD14*, which encodes a receptor for bacterial LPS, influence how individuals respond to endotoxin-rich environments.^136^ Children with certain CD14 genotypes experience stronger protection against atopy when raised on farms or when consuming unpasteurized farm milk,^137^ reflecting the capacity of microbial and dietary inputs to modulate genetic susceptibility.^138^ Another novel research area is how the interaction between maternal vitamin D pathway genes and vitamin D levels during pregnancy affects asthma outcomes in offspring.^139^ Polymorphisms in the vitamin D receptor or vitamin D-binding protein genes appear to determine to what extent maternal vitamin D deficiency increases the risk of childhood asthma.^140^

Even in pharmacotherapy, gene–environment interactions play a role. Cigarette smoke exposure can reduce the effectiveness of ICS by triggering oxidative stress that suppresses HDAC2 activity, a key component of steroid signaling.^141^ Specific glucocorticoid receptor polymorphisms might further predispose smokers with asthma to steroid resistance, illustrating how genetics, environment, and drug response intersect in determining disease control.^142^

Ethnic differences in asthma prevalence also reflect, in part, the complex interplay between genetic background and environmental exposures. Populations of African ancestry, for example, experience a disproportionately high asthma burden in Western countries.^143^ This disparity likely results from ancestry-specific genetic variants interacting with urban environmental conditions such as air pollution, allergen exposure, and psychosocial stress.^144^ One such variant occurs in the *PYHIN1* gene, identified as an asthma risk locus in genome-wide studies of African Americans but rare among Europeans.^145^ The impact of this variant may be heightened by urban exposures that amplify inflammatory responses.^146^ Socioeconomic disadvantage, which encapsulates multiple environmental stressors, can also modify biological vulnerability.^143^ Children living in crowded housing or polluted neighborhoods and exposed to chronic stress may develop more severe asthma when carrying certain stress-response genotypes, such as variants in *ADRβ2* or *ACE*.^147,148^

From a translational standpoint, recognizing gene–environment interactions can improve risk stratification and suggest interventions.^149^ If a child is known to carry high-risk alleles that make them vulnerable to pollution’s effects, one might prioritize interventions such as moving them away from high-traffic areas or using indoor air filters.^150^ A toddler with eczema who carries a high-risk *IL33* genotype might warrant special precautions during viral seasons, including early antiviral therapy or preventive biologics against RSV. Advances in this field are already guiding precision prevention trials. High-risk infants, identified by family history or genetic screening, are being studied for interventions such as probiotic supplementation or immunomodulators derived from farm dust that aim to replicate the protective effects of rural microbial exposure.^151^

Gene–environment interactions weave a complex tapestry of asthma risk and expression. Asthma can no longer be viewed as solely a genetic disorder or purely an environmental disease; it is a product of their combined effects. Future risk models may integrate genetic risk scores with personalized environmental exposure profiles to offer individualized asthma risk assessments. In terms of treatment, this could extend into the realm of pharmacogenomics—for instance, specific genotypes of the β_2_-adrenergic receptor (*ADRB2*) influence responses to salbutamol, while variations in 5-lipoxygenase (*ALOX5*) may affect sensitivity to leukotriene modifiers.^152^ By considering these interactions, clinicians can move toward the goal of “delivering the right intervention to the right patient at the right time”—effectively embodying the bench-to-bedside translation of asthma science.^149^ Integrating this understanding allows intervention strategies to shift from broad, population-level approaches to more precise, individualized prevention and treatment. Crucially, it reaffirms the central role of environmental interventions—ensuring clean air, preventing infections, supporting smoking cessation, and maintaining a healthy microbiome are not merely adjunctive measures but are key to asthma control, particularly for those with genetic predispositions.^153^

The rising prevalence of asthma over the last half-century underscores the potent influence of environmental change. Early-life exposures are particularly crucial in shaping immune development toward allergic (atopic) or nonallergic trajectories. Having explored the origins of asthma susceptibility, attention now turns to the pathophysiological and immunological mechanisms that operate once the disease is established, revealing how these genetic and environmental forces translate into the airway inflammation and remodeling that define asthma.

## Pathophysiology and immunological mechanisms

Asthma pathophysiology centers on an inflammatory cascade that injures the airway lining and a remodeling process that alters its structure. These processes lead to variable airflow obstruction caused by bronchospasm and mucus accumulation and to bronchial hyperresponsiveness, the exaggerated tendency of airways to constrict in response to stimuli. Inflammation sensitizes the airway nerves and smooth muscle, while geometric changes in thickened airway walls further enhance constriction according to Laplace’s law.^154^ These features result from the interplay among immune cells, structural cells such as epithelial cells, airway smooth muscle, fibroblasts, and neural inputs within the airway. This section delineates the main pathophysiological changes in asthmatic airways and the cellular and molecular networks driving them.

### Airway inflammation, remodeling, and mucus impaction

Asthma is defined by chronic inflammation of the airways, which progressively leads to structural remodeling and excessive mucus production. These processes underlie the key clinical features of the disease: airway hyperresponsiveness and variable airflow obstruction. Inflammation is not static and can intensify during exacerbations triggered by allergens or infections, but even in periods of apparent stability, the airway structure and function remain abnormal compared with healthy individuals.

A central feature of asthma is epithelial damage. The airway epithelium, normally a protective barrier, becomes fragile and disrupted. Biopsy studies have revealed epithelial cell shedding (Creola bodies seen in sputum) and loss of tight junction integrity, which increase permeability to allergens and pollutants.^155^ Injured epithelial cells release “danger signals,” notably alarmins such as TSLP, IL-33, and IL-25, which rapidly activate downstream immune pathways.^156^ This initiates a self-perpetuating cycle: environmental exposures damage the epithelium, the damaged epithelium secretes alarmins, and recruited immune cells further aggravate epithelial injury.^157^

**Fig. 3:**
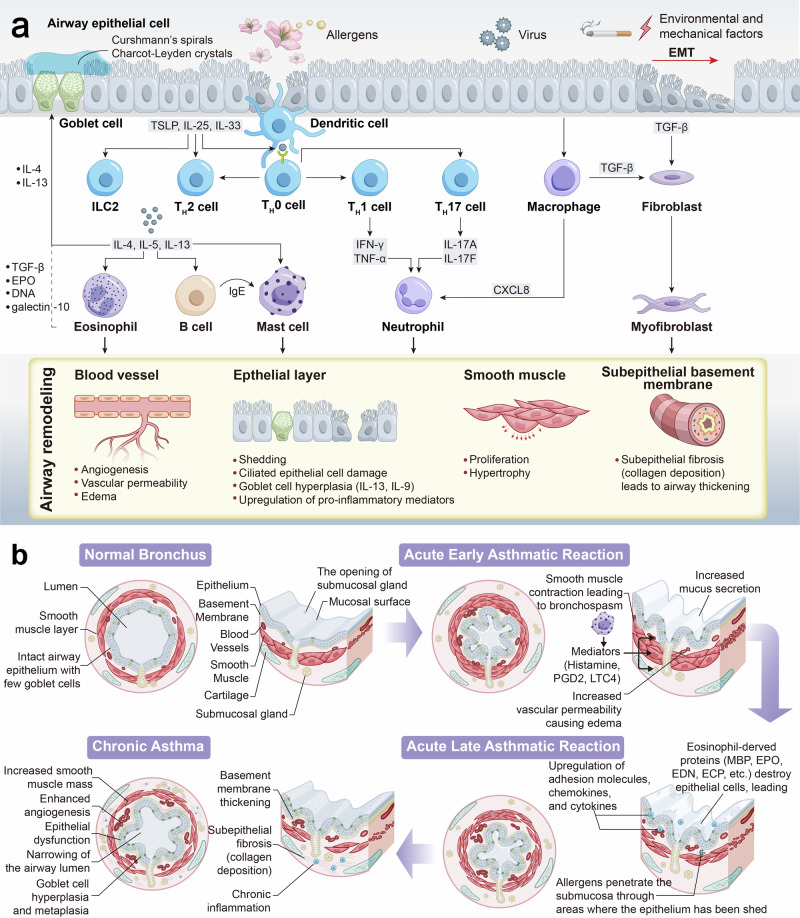
Mechanisms and histopathological progression of airway remodeling in asthma. **a** Airway epithelial injury and immune activation drive remodeling through cytokine- and growth factor–mediated changes in the epithelium, subepithelial matrix, smooth muscle, and vasculature. **b** Simulated anatomical illustrations show representative structural alterations from the normal bronchus through acute early and late asthmatic reactions to chronic asthma, characterized by persistent inflammation, mucus hypersecretion, smooth muscle hypertrophy, and fixed airway narrowing. The panels are conceptual and intended to depict typical pathological evolution rather than universal stages observed in every patient. This figure was painstakingly hand-drawn by the author(s) using Adobe Illustrator, stroke by stroke

Airway inflammation in asthma is typically diffuse but can appear patchy, involving both the large (central) and small (peripheral) airways. In classic allergic asthma, eosinophilic inflammation predominates, primarily affecting the bronchial walls from central bronchi to terminal bronchioles. In contrast, neutrophilic asthma is dominated by neutrophil infiltration. Regardless of the inflammatory type, chronic untreated inflammation eventually leads to airway remodeling—permanent structural alterations that contribute to airflow limitation and airway hyperresponsiveness (Fig. 3).

Key histopathological features of airway remodeling include the following:

#### Subepithelial fibrosis

The basement membrane appears thickened due to collagen and extracellular matrix deposition beneath it. This is visible on pathology and even via airway imaging and reflects longstanding injury and repair processes. Fibroblasts and myofibroblasts, activated by transforming growth factor β (TGF-β) and other mediators released from eosinophils and epithelial cells, produce collagen types I and III, leading to a stiffer, less compliant airway wall.^158^

#### Airway smooth muscle (ASM) hypertrophy and hyperplasia

Asthmatic airways contain a significantly increased smooth muscle mass resulting from both hypertrophy (enlarged cell size) and hyperplasia (increased cell number). This expanded ASM layer contributes to airway narrowing and hypercontractility. Cytokines such as IL-13,^159^ IL-11, and growth factors such as TGF-β, PDGF, and IGF-1 promote ASM growth. Moreover, ASM cells actively participate in inflammation by secreting cytokines and matrix components.^160^

#### Mucus gland hyperplasia and goblet cell metaplasia

Chronic stimulation by IL-13 and epidermal growth factor receptor (EGFR) signaling leads to enlargement of submucosal mucus glands in larger airways and proliferation of goblet cells in smaller airways.^161^ This shift from ciliated to mucus-secreting epithelium produces excessive, highly viscous mucus rich in MUC5AC and cellular DNA.^162^ Asthmatic mucus is rich in high-molecular-weight mucins, mainly MUC5AC from goblet cells and MUC5B from submucosal glands. IL-13 is a potent driver of goblet cell metaplasia and MUC5AC production. During acute exacerbations, viscous mucus can form plugs that obstruct airways, leading to life-threatening airflow limitation. Autopsy studies of fatal asthma consistently reveal extensive mucus plugging in segmental and subsegmental bronchi.^163^

Advances in imaging have enabled in vivo visualization of mucus obstruction. High-resolution computed tomography (CT) reveals tube-like opacities representing mucus-filled bronchi. In severe asthma, a higher mucus plug score on CT correlates with poorer lung function and more frequent exacerbations.^164^ These plugs often localize to lower lobes due to gravity.^165^ Encouragingly, biologic therapies targeting upstream mediators can reduce mucus burden. Clinical trials of tezepelumab, an anti-TSLP antibody, have shown reductions in mucus plug scores on CT along with improvements in lung function,^166^ suggesting that blocking epithelial cytokine signaling can mitigate mucus overproduction by dampening IL-13 and related type 2 pathways.

#### Angiogenesis

Asthmatic bronchial biopsies demonstrate increased vascularity within the airway wall, largely driven by vascular endothelial growth factor (VEGF).^167^ These new, leaky vessels exacerbate airway edema and promote inflammatory cell recruitment.

#### Cartilage changes

In severe cases, even cartilage in larger airways can be thinned or damaged.^168^

These remodeling changes stiffen the airway wall and narrow the lumen. Small airways, typically less than 2 mm in diameter, are particularly vulnerable because they lack supportive cartilage.^169^ Fibrosis, smooth muscle thickening, and mucus plugging can obstruct the lumen, contributing significantly to airflow limitation. Dysfunction in these “quiet zones” correlates closely with disease severity, often even when spirometry values appear normal.^170^ Over time, such remodeling can produce fixed airflow obstruction resembling COPD; up to 30–50% of patients with long-standing asthma develop some degree of irreversible limitation. Notably, remodeling begins early in life—subepithelial fibrosis and ASM thickening have been observed in young children with recurrent wheezing.^171^

New imaging and physiological techniques have deepened the understanding of airway remodeling and obstruction. Parametric response mapping (PRM) of CT scans is an advanced technique that differentiates functional small airway disease (air trapping) from emphysema. PRM studies show that many asthmatics exhibit significant functional small airway disease (fSAD) even when FEV_1_ is normal, and the extent of fSAD correlates with poor disease control.^172^ Hyperpolarized gas MRI (using ^129^Xe or ^3^He) further reveals patchy ventilation defects corresponding to transient airway closure—these defects worsen during bronchoconstriction and improve after bronchodilator use.^173,174^ Similarly, impulse oscillometry (IOS) provides sensitive, noninvasive assessment of small airway resistance and reactance, detecting dysfunction even when standard spirometry is normal.^175^ These methods are emerging as valuable tools for monitoring disease progression and treatment response.

### Cellular participants and cytokine networks

**Fig. 4:**
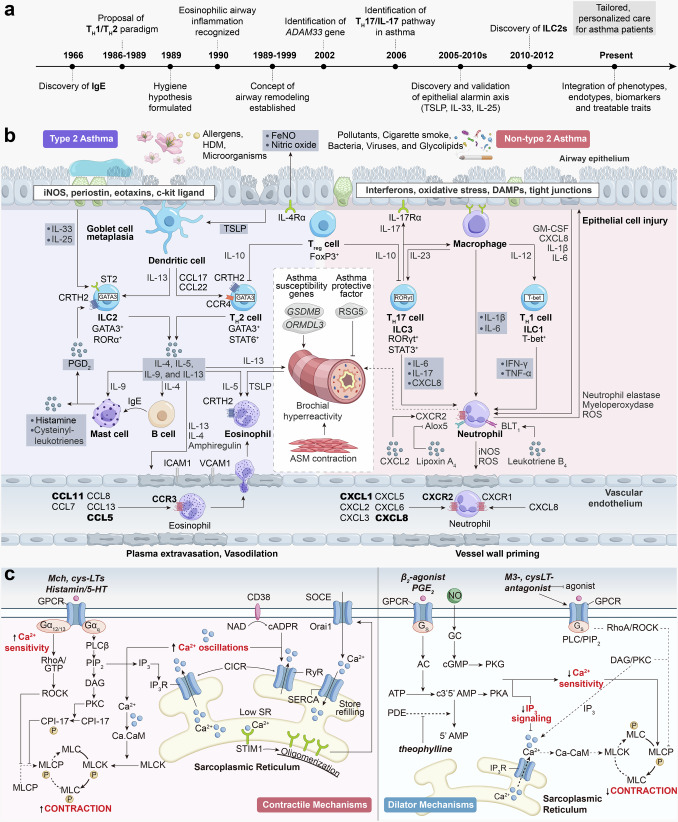
Historical evolution, immunologic mechanisms, and regulation of airway smooth muscle tone in asthma. **a** Chronological milestones in asthma research from immunologic discovery to precision endotyping, illustrating major conceptual and molecular breakthroughs that shaped current understanding. **b** Integrated immunologic framework of asthma pathogenesis. Type 2–dominant inflammation is initiated by epithelial alarmins (TSLP, IL-33, IL-25) that activate dendritic cells, Th2 lymphocytes, ILC2s, eosinophils, and mast cells, driving IL-4/IL-5/IL-13–mediated eosinophilic inflammation, mucus hypersecretion, and airway hyperresponsiveness. In contrast, non–type 2–dominant pathways involve IL-17, IL-6, IFN-γ, and TNF-α signaling, promoting neutrophilic inflammation, oxidative stress, and corticosteroid insensitivity. **c** Regulation of airway smooth muscle (ASM) contraction and relaxation. Contractile agonists such as methacholine, histamine and leukotrienes trigger G_q_-coupled receptor activation, phospholipase C-dependent generation of IP_3_ and DAG, and Ca^2+^ release from the sarcoplasmic reticulum, leading to myosin light chain (MLC) phosphorylation via the Ca^2+^/calmodulin–MLCK pathway. RhoA/ROCK and PKC-CPI-17 signaling sustain contraction by inhibiting MLC phosphatase. Relaxation is mediated by β_2_-adrenergic and nitric oxide (NO) signaling: cAMP–PKA and cGMP–PKG pathways reduce cytosolic Ca^2+^, enhance sarcoplasmic reticulum reuptake, and promote MLC dephosphorylation. Coordinated regulation of these cascades maintains airway tone and responsiveness. This figure was hand-crafted by the author(s) using Adobe Illustrator. Figure 4b was adapted from our previous work (Xie et al., 2024, Front. Immunol.)^157^, with extensive modifications

The asthmatic inflammatory milieu is sustained by a complex cast of immune and structural cells orchestrated by a network of cytokines and lipid mediators. Traditionally, asthma has been viewed as a Th2 cell–driven disease. In many patients, CD4^+^ Th2 lymphocytes play a central role by secreting the canonical type 2 cytokines IL-4, IL-5, and IL-13, which drive IgE production, eosinophilia, and goblet cell hyperplasia, respectively. It is now evident, however, that a wide array of both innate and adaptive immune cells contribute to the asthmatic response, particularly in non-T2 phenotypes (Fig. 4a, b).^176^

Key cellular players include:

#### Dendritic cells (DCs)

DCs are antigen-presenting cells strategically positioned in the airway epithelium and submucosa. Upon inhalation of allergens or viruses, they capture, process, and present antigens to naïve T cells in the draining lymph nodes.^177^ Pulmonary DCs, conditioned by epithelial cytokines such as TSLP, IL-25, and GM-CSF, promote the differentiation of naïve T cells into Th2 effectors. TSLP is a potent activator of myeloid DCs, upregulating OX40L on DCs, which drives Th2 polarization.^178,179^ Some DC subsets also produce IL-25 and IL-6, which can favor Th17 responses in certain contexts.^180^ DCs thus serve as initiators of the adaptive immune cascade in allergic asthma.

#### T lymphocytes

CD4^+^ helper T cells coordinate much of the immune response. In classical asthma, Th2 cells dominate and release IL-4, IL-5, and IL-13, which drive IgE class switching, eosinophil recruitment, and goblet cell hyperplasia with bronchoconstriction, respectively. Th2 cells can be allergen-specific, forming memory populations that react rapidly upon re-exposure. In some patients, Th17 cells contribute to disease by secreting IL-17A, IL-17F, and IL-22. These cytokines recruit neutrophils and enhance barrier defense but also promote airway remodeling. IL-17 levels are elevated in the sputum of patients with severe steroid-resistant asthma, although IL-17 blockade has not produced clinical benefit,^181^ suggesting that Th17 cells may be secondary amplifiers rather than primary initiators.

Regulatory T cells (Tregs), characterized by FoxP3 expression, are essential for maintaining tolerance and suppressing effector T-cell activity.^182^ Asthmatic individuals often exhibit reduced numbers or impaired function of Tregs.^183^ Allergen immunotherapy has been shown to increase allergen-specific Tregs, correlating with clinical improvement. In severe asthma, some Tregs lose suppressive capacity or convert to Th2-like phenotypes under the influence of local cytokines. Experimental strategies to enhance Treg function, such as low-dose IL-2 administration or adoptive Treg transfer, are under investigation.^184^

#### Innate lymphoid cells (ILCs)

Among innate immune populations, group 2 ILCs (ILC2s) have emerged as important players in asthma.^185^ They are considered innate counterparts of Th2 cells, lacking antigen-specific receptors but capable of producing large quantities of IL-5, IL-13, and some IL-9 in response to epithelial-derived alarmins such as TSLP, IL-33, and IL-25 and neuropeptides. ILC2s are resident in lung tissue and are activated within hours during asthma exacerbations, particularly in response to viral infections or non-allergen triggers. They can cause rapid eosinophil influx and bronchospasm independent of T cells.

ILC2s express receptors such as ST2 (for IL-33) and IL-17RB (for IL-25) and, interestingly, the neuromedin U receptor (NMUR1).^186^ Neuromedin U, released from airway sensory neurons, binds to NMUR1 on ILC2s and triggers IL-5 and IL-13 secretion, forming a link between neural signaling and type 2 inflammation.^187^ In nonatopic asthma, ILC2s activated by viruses or pollutants through IL-33 may drive eosinophilic inflammation when Th2 cells are scarce. Corticosteroid therapy reduces ILC2 populations expressing NMUR1, suggesting that part of its benefit derives from dampening ILC2 activation. Other ILC subsets, such as ILC1s and ILC3s, also exist, and there is evidence of plasticity between them. Under IL-1 and IL-23 stimulation, some ILC2s can acquire IL-17–producing capacity, creating intermediate “ILC2/3” phenotypes observed in severe asthma.^188^

#### Eosinophils

Eosinophils, a hallmark of allergic asthma, originate in the bone marrow and migrate to the lungs under the influence of IL-5 and eotaxins such as CCL11. They release cytotoxic granules containing major basic protein and eosinophil peroxidase, along with lipid mediators such as leukotrienes that damage the epithelium and provoke bronchoconstriction and mucus secretion. Through TGF-β production, eosinophils also promote tissue remodeling and fibrosis. Eosinophil counts in sputum correlate with exacerbation risk and responsiveness to ICS. Anti–IL-5 therapies that deplete eosinophils have confirmed their central role in driving exacerbations, although eosinophils are likely downstream effectors of Th2 and ILC2 signaling rather than primary initiators.^189,190^

#### Mast cells

Mast cells reside within the airway wall and often infiltrate the smooth muscle layer in asthmatics.^191^ They carry IgE bound to FcεRI receptors and degranulate upon allergen-induced IgE crosslinking. This process releases histamine, prostaglandin D_2_ (PGD_2_), leukotriene C_4_/D_4_, and tryptase. Histamine and cysteinyl leukotrienes are potent bronchoconstrictors and vasodilators that induce edema. Mast cell-derived tryptase can also activate proteinase-activated receptors on smooth muscle cells, leading to their contraction. Moreover, sustained activation of mast cells and their released mediators plays a crucial role in airway remodeling; specifically, tryptase and chymase promote both fibroblast proliferation and smooth muscle cell growth/hyperplasia. PGD_2_, a major mast cell–derived prostanoid, signals through the CRTH2 receptor (DP2) on Th2 cells, eosinophils, and ILC2s, amplifying type 2 inflammation.^192^ Antagonism of CRTH2, as tested with fevipiprant, aimed to disrupt this circuit, although phase III trials did not achieve clinical success.^193^ In aspirin-exacerbated respiratory disease (AERD), mast cells overproduce cysteinyl leukotrienes and PGD_2_ because COX-1 inhibition shifts arachidonic acid metabolism toward the leukotriene pathway. This imbalance explains the intense eosinophilic inflammation and persistent bronchoconstriction in AERD – high PGD_2_ via CRTH2 recruits more Th2/eosinophils, while high leukotrienes cause persistent bronchoconstriction. Aspirin desensitization and leukotriene modifiers mitigate this by restoring some metabolic balance.

#### Neutrophils

Neutrophils dominate in some severe or steroid-refractory forms of asthma, especially those associated with smoking history, obesity, or frequent exacerbations. They release proteases such as elastase and MMP-9, as well as reactive oxygen species that perpetuate inflammation and tissue damage. Neutrophil extracellular traps (NETs) are also found in asthmatic airways, where their DNA content thickens mucus and sustains inflammation.^194^ IL-8 (CXCL8) and GM-CSF from airway epithelial cells recruit and prolong neutrophil survival.

Th17 cells and ILC3s maintain neutrophilic inflammation via IL-17 and IL-22 signaling. Corticosteroids often fail to control this pattern and may even extend neutrophil lifespan by inhibiting apoptosis. Macrolide antibiotics such as azithromycin can reduce exacerbation frequency in neutrophil-predominant asthma, likely through anti-inflammatory effects and modulation of the airway microbiome.^22^ However, clinical trials targeting IL-17 have not demonstrated substantial benefit, suggesting that neutrophils may mark a complex, treatment-refractory endotype rather than serve as direct effectors or that redundancy in neutrophil recruitment pathways complicates single-cytokine targeting.

#### Macrophages

Airway macrophages, either tissue-resident or monocyte-derived, adopt distinct polarization states. In allergic asthma, IL-4 and IL-13 drive polarization toward the “M2” phenotype, which produces chemokines such as CCL17 and CCL22 that attract Th2 cells and promote fibrotic remodeling via TGF-β. Macrophages also generate lipid mediators: leukotriene B_4_, which attracts neutrophils, and prostaglandin E_2_, which exerts protective bronchodilatory effects. The balance of macrophage mediators might influence exacerbation severity. Although macrophages normally aid in debris clearance, their inflammation-resolving functions appear impaired in asthma. Therapeutic strategies that repolarize macrophages toward a pro-resolving state through pathways such as PPAR-γ or AMPK activation are under investigation.

#### Airway structural cells

Structural cells, including epithelial cells, smooth muscle cells, and fibroblasts, actively shape the inflammatory milieu. Epithelial cells release alarmins such as TSLP, IL-33, and IL-25, which initiate type 2 immunity. Airway smooth muscle cells secrete IL-6, GM-CSF, and chemokines that recruit immune cells, functioning not only as contractile elements but also as amplifiers of inflammation. Fibroblasts respond to IL-13 and TGF-β by producing periostin, a matricellular protein associated with airway remodeling and used as a biomarker for Th2-high disease. Elevated periostin levels often predict a better response to IL-13-targeted therapies such as lebrikizumab.

**Fig. 5:**
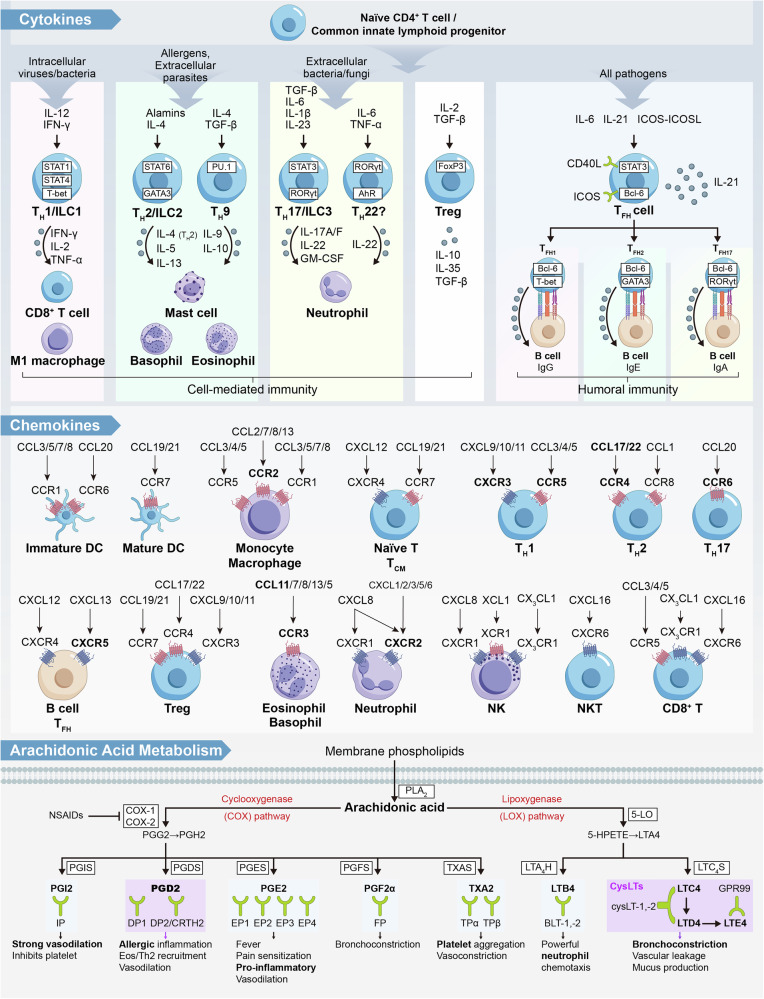
Cytokine, chemokine, and arachidonic acid pathways in asthma. Asthmatic immunity is shaped by multiple T-cell and innate lymphoid cell subsets that, under distinct stimuli and transcriptional programs, produce cytokines that drive airway inflammation and tissue responses. The recruitment of immune cells into the airways depends on a range of chemokine–receptor axes involving dendritic cells, macrophages, diverse T-cell subsets, B cells, and granulocytic and innate effector cells. Arachidonic acid metabolism generates prostaglandins, leukotrienes, lipoxins, and related lipid mediators that signal through specific receptors to modulate inflammatory amplification, mucus production, and airway smooth muscle tone, together forming a key network influencing asthma phenotypes. This figure was drawn manually by the author(s) using Adobe Illustrator

The cytokine networks in asthma are extensive, but some key pathways include (Fig. 5):

#### IL-4/IL-13 pathway

IL-4 drives Th2 differentiation and IgE class switching in B cells, while IL-13 mediates mucus production and bronchial hyperresponsiveness.^195^ Both signal through the IL-4Rα receptor, activating the JAK-STAT6 pathway that controls the transcription of type 2–associated genes. IL-4/IL-13 can also activate the insulin receptor/IRS-2 pathway in epithelial cells, leading to goblet cell hyperplasia. Because of their central role, dual blockade of IL-4 and IL-13 with agents such as dupilumab has shown broad efficacy in T2-high asthma, reducing exacerbations and improving lung function across both allergic and eosinophilic subtypes.

#### IL-5 pathway

IL-5 is essential for eosinophil development, activation, and survival. Produced by Th2 cells, ILC2s, and mast cells, it maintains eosinophil production in the bone marrow and primes their effector functions. Neutralization of IL-5 with mepolizumab or reslizumab or blockade of its receptor with benralizumab causes near-complete eosinophil depletion and effectively prevents exacerbations in eosinophilic asthma.^196^ These findings confirm eosinophils as key effectors, although residual airway dysfunction suggests that additional pathways also contribute.

#### IL-17/Th17 pathway

A subset of patients exhibits elevated IL-17A, IL-17F, and IL-22, produced by Th17 and possibly ILC3 cells. These cytokines recruit neutrophils and promote steroid resistance.^197^ IL-17 also stimulates epithelial cells to produce GM-CSF and IL-8, perpetuating inflammation.^198^ Despite this, IL-17 blockade has shown limited clinical benefit, indicating redundancy among neutrophil-recruiting pathways. Some evidence even suggests that IL-17 may suppress Th2 responses under specific conditions, making its role in human asthma complex and context dependent.^199,200^

#### TNF-α and IL-1

These pro-inflammatory cytokines rise during severe exacerbations and in subsets of refractory asthma. They promote neutrophil recruitment, adhesion molecule expression, and tissue remodeling. Clinical trials of TNF-α blockers have yielded inconsistent results and raised safety concerns,^201,202^ while IL-1β appears to be particularly relevant in obesity-related or occupational asthma.

#### TGF-β and other growth factors

TGF-β drives airway remodeling by inducing fibroblast-to-myofibroblast differentiation, smooth muscle proliferation, and extracellular matrix deposition.^203^ It is released by epithelial cells, macrophages, eosinophils, and platelets. Elevated airway TGF-β correlates with fibrosis and fixed airflow obstruction. Although no direct anti-TGF-β therapies are yet in routine use, targeting eosinophils with IL-5 inhibitors may indirectly lower TGF-β activity and remodeling.

A special mention is warranted for lipid mediators derived from arachidonic acid, as they form a crucial inflammatory network in asthma (Fig. 5). In asthma, the balance is tipped toward bronchoconstrictive and proinflammatory eicosanoids:

#### The 5-lipoxygenase pathway

This pathway generates **leukotrienes**. LTB_4_ acts as a neutrophil chemoattractant, while cysteinyl leukotrienes (LTC_4_, LTD_4_, LTE_4_) induce bronchoconstriction, mucus production, and vascular permeability through CysLT_1_ receptors on airway smooth muscle. Leukotriene receptor antagonists such as montelukast alleviate symptoms by blocking this receptor.^204^ Asthmatic patients exhale increased LTE_4_ during attacks, and in AERD, the 5-LO and LTC_4_ synthase pathways are overactive, producing excessive leukotrienes. Both leukotriene inhibitors and aspirin desensitization reduce this overproduction.

#### The cyclooxygenase pathway

This pathway produces **prostaglandins** and **thromboxanes**. PGD_2_, mainly released from mast cells, attracts Th2 cells, ILC2s, and eosinophils through CRTH2 signaling, thereby amplifying type 2 inflammation.^205^ It is also a key mediator in AERD. PGD_2_ also causes bronchoconstriction via DP1 receptors.^206^ In contrast, prostaglandin E2 (PGE_2_) has bronchodilatory and anti-inflammatory properties through EP2/EP4 receptor signaling. Many asthmatics exhibit relative PGE_2_ deficiency. In aspirin-sensitive individuals, COX-1 inhibition further reduces PGE_2_ and removes its restraint on the 5-LO pathway, allowing excessive leukotriene generation. Thromboxane A_2_, another COX product, promotes bronchoconstriction and platelet aggregation and may contribute to aspirin-induced reactions in susceptible patients.

#### The lipoxin pathway

Lipoxins such as LXA_4_ serve as endogenous pro-resolving mediators that limit inflammation and promote tissue recovery. These molecules are often deficient in asthma, skewing the balance toward persistent inflammation.^207^ Synthetic analogs of lipoxins and related mediators such as resolvins and protectins are being explored as therapies aimed at restoring resolution rather than merely suppressing inflammation.^208,209^ This could help quell the chronicity of asthma inflammation and perhaps promote true disease remission, which is a goal on the horizon.

Collectively, asthma inflammation is a coordinated attack involving multiple immune cells that communicate via cytokines, chemokines, and lipid mediators within a network. The type 2 cytokine triad—IL-4, IL-5, and IL-13—remains central to asthmatic inflammation. IL-4 drives Th2 differentiation and IgE production; IL-5 governs eosinophil maturation and survival; and IL-13 promotes mucus secretion, airway hyperresponsiveness, and B-cell activation. Blocking these pathways with monoclonal antibodies has led to significant clinical improvement in type 2–high asthma. In other endotypes, cytokines such as IFN-γ and IL-17 dominate. IL-17 promotes neutrophil recruitment and contributes to steroid resistance and airway remodeling. GM-CSF and IL-3 enhance the persistence of eosinophils and basophils. Epithelial cytokines—TSLP, IL-33, and IL-25—act as upstream activators of type 2 immunity. TNF-α and IL-1β are elevated in severe, neutrophilic asthma, promoting steroid resistance and sustaining inflammation, although therapeutic targeting of these cytokines has shown limited success.

Immune cells interact through tightly linked feedback loops. Mast cells and airway smooth muscle maintain a reciprocal relationship: mast cell mediators induce muscle contraction and proliferation, while smooth muscle–derived stem cell factor (SCF) supports mast cell survival.^191^ Eosinophils and epithelial cells form another circuit, where eosinophil-derived TGF-β stimulates fibrosis and epithelial IL-13 drives eotaxin release, recruiting more eosinophils. Neutrophil elastase disrupts epithelial barriers and triggers further IL-8 secretion, perpetuating neutrophilic cycles. Macrophages influence T-cell differentiation through IL-12 and IL-10, but in allergic asthma, their regulatory capacity often becomes overwhelmed.

### Signal transduction and metabolic control

**Fig. 6:**
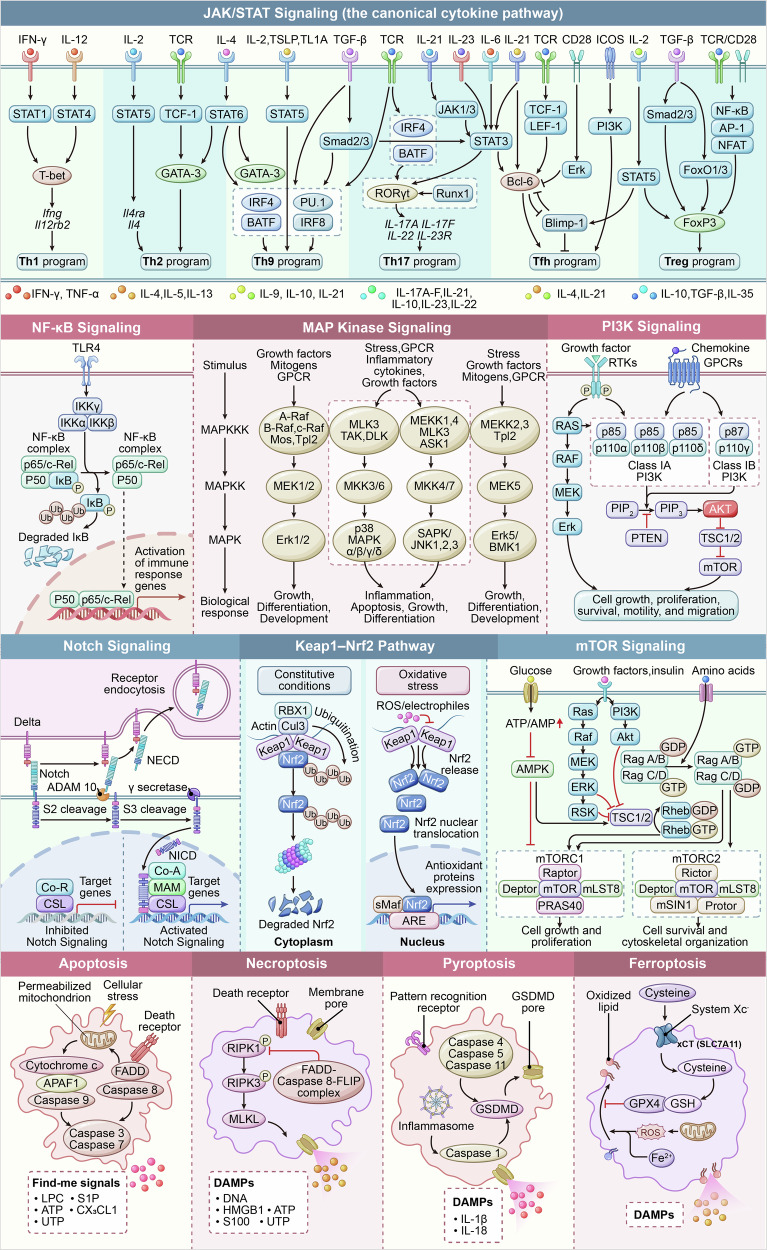
Intracellular signaling pathways involved in asthma pathobiology. Key cytokine-responsive cascades include JAK–STAT signaling activated by IL-4, IL-5, IL-13, and TSLP; NF-κB transcriptional programs driving pro-inflammatory mediator production; MAP kinase pathways regulating epithelial stress responses and airway hyperresponsiveness; PI3K–AKT signaling contributing to survival, proliferation, and corticosteroid resistance; Notch signaling controlling epithelial differentiation and immune polarization; Keap1–Nrf2 oxidative-stress pathways governing antioxidant defenses; and mTOR signaling shaping cellular metabolic and anabolic activity. Programmed cell-death pathways—apoptosis, necroptosis, pyroptosis, and ferroptosis—further modulate epithelial injury, mucus pathology, and chronic airway remodeling. Although not exhaustive, these interconnected modules outline the layered molecular architecture underlying asthma heterogeneity. Every line and label in this figure was manually placed by the author(s) using Adobe Illustrator, without reliance on AI image generators

The engagement of receptors on the surface of immune and structural cells in asthma initiates a cascade of intracellular signaling pathways. These interconnected networks regulate cellular activation, cytokine gene transcription, proliferation, and survival, thereby sustaining the chronic inflammation and airway hyperreactivity characteristic of the disease (Fig. 6). Several major signaling mechanisms have been identified.^210^

#### JAK–STAT pathway

Many cytokines central to asthma pathogenesis signal through the Janus kinase (JAK)–signal transducer and activator of transcription (STAT) pathway.^211^ IL-4 and IL-13 engage the type I or type II IL-4 receptor, activating JAK1, JAK3, and Tyk2, which subsequently phosphorylate STAT6.^212^ Phosphorylated STAT6 forms dimers that translocate to the nucleus and drive the transcription of key Th2-associated genes, including IL-4, IL-13, the chemokine CCL26 (eotaxin-3), and the IgE heavy-chain gene in B cells.^213^ The importance of this pathway is underscored by the observation that STAT6-deficient mice exhibit markedly attenuated asthmatic features, lacking Th2 differentiation and eosinophilia.^214^ IL-5 signals through the IL-5 receptor, utilizing JAK2 and STAT5, while TSLP signals through the TSLPR–IL-7Rα complex, activating JAK1, JAK2, and STAT5.^215^ JAK1 and JAK3 thus represent critical nodes in type 2 inflammation. This understanding has driven the development of JAK inhibitors as potential asthma therapeutics.^216^ Because systemic JAK inhibition can cause adverse effects, recent efforts have focused on inhaled JAK1-selective inhibitors such as AZD0449 and AZD4604/lonadamocitinib, designed to suppress airway cytokine signaling locally while minimizing systemic exposure.^217^ Preliminary phase I data have shown that inhaled JAK1 inhibitors can achieve lung pharmacodynamic effects by reducing biomarker gene expression without detectable systemic accumulation.^218^ These compounds could, if effective, simultaneously attenuate multiple cytokine pathways, including IL-4, IL-5, IL-13, IL-33, and TSLP, offering a comprehensive approach to severe asthma with overlapping inflammatory mechanisms.^216^

#### NF-κB pathway

Nuclear factor kappa B (NF-κB) is a master transcriptional regulator of pro-inflammatory gene expression.^219^ It is activated by numerous stimuli in both immune and structural cells, including engagement of Toll-like receptors by pathogens, activation of IL-1β and TNF receptors, and exposure to oxidative stress. In resting cells, NF-κB dimers such as p50/p65 are retained in the cytoplasm through binding to inhibitory IκB proteins.^220^ Activation of the upstream IκB kinase (IKK) complex leads to phosphorylation and degradation of IκB, allowing NF-κB to translocate into the nucleus and initiate transcription of inflammatory mediators, including IL-8, TNF-α, GM-CSF, inducible nitric oxide synthase (iNOS), and many chemokines.^219^ In asthma, NF-κB is persistently active in airway macrophages and epithelial cells due to ongoing stimulation by allergens, microbial products, and oxidative insults. This pathway contributes to both neutrophilic inflammation and chronic persistence of the disease.^221^ Corticosteroids partly suppress NF-κB activity by inducing IκB expression, but oxidative stress can reduce HDAC2 activity, leading to steroid resistance.^222^ Activation of AMP-activated protein kinase (AMPK) by adiponectin can inhibit NF-κB and iNOS,^223^ which may explain the stronger NF-κB–driven inflammation seen in obese patients with low adiponectin levels.^224^ Because NF-κB signaling is ubiquitous, direct pharmacologic inhibition is limited by toxicity. Indirect approaches, such as antioxidant therapies to limit oxidative NF-κB activation or blockade of upstream cytokines such as IL-1 and TNF, may offer safer modulation. In this context, inhibition of p38 MAPK, which partially regulates NF-κB activation, has shown promise in reducing inflammatory markers, although liver toxicity has constrained clinical application.

#### Mitogen-activated protein kinase (MAPK) pathways

The MAPK family, comprising p38, ERK, and JNK subfamilies, is activated by various stress signals and cytokines.^225,226^ In asthma, p38 MAPK is activated in airway epithelial cells and macrophages in response to stimuli such as LPS and osmotic stress, inducing the production of TNF, IL-1β, and chemokines. ERK1/2 responds to growth factors and contributes to airway smooth muscle (ASM) proliferation and goblet cell hyperplasia.^227^ JNK activation by IL-13 and IL-17 in ASM alters integrin expression and may enhance contractility. MAPKs also modulate transcription factors through serine phosphorylation; both p38 and ERK can enhance STAT6 transcriptional activity, thereby amplifying IL-13–mediated responses. Inhibiting MAPKs could therefore reduce IL-13 effects by blocking STAT6 activation. Clinical trials with p38 inhibitors have demonstrated decreases in sputum neutrophils and inflammatory mediators,^228^ but clinical benefits were modest, and hepatotoxicity limited further development. Current strategies are exploring inhaled p38 inhibitors such as losmapimod formulations to localize the therapeutic effect within the airways.^229^

#### PI3K/AKT pathway

The phosphoinositide 3-kinase–AKT pathway, particularly the δ isoform of PI3K, is frequently overactive in asthma.^230^ PI3Kδ is activated by various receptors, including the IL-5 receptor on eosinophils and several chemokine receptors, leading to AKT (protein kinase B) phosphorylation and downstream signaling that promotes survival, metabolism, and migration. In severe asthma, enhanced PI3K/AKT signaling contributes to corticosteroid insensitivity through HDAC2 inactivation. Inhaled PI3Kδ inhibitors such as nemiralisib were therefore developed to restore steroid responsiveness and dampen inflammation.^231^ Although these agents demonstrated target engagement by reducing phosphorylated AKT in sputum cells, clinical outcomes such as lung function and exacerbation rates did not improve significantly.^231^ These results suggest that inhibition of PI3K alone may be insufficient to reverse established steroid resistance or that dosing and tolerability issues, including cough associated with inhaled delivery, limited efficacy. PI3Kδ inhibition may nonetheless prove useful in specific phenotypes, such as virus-induced or neutrophilic asthma, where the pathway is more active.

#### Innate inflammasomes and IL-1

Non-eosinophilic asthma may involve innate immune activation via inflammasomes in airway cells. The NLRP3 inflammasome, triggered by cellular stress or danger signals such as ATP or silica, leads to the release of IL‑1β and IL‑18. Elevated IL‑1β has been observed in neutrophilic asthma, where it can promote Th17 responses and enhance neutrophil survival.^232^ A recent review noted that inflammasome-related genes and signatures including *IL1B* and *CASP1* are upregulated in some patients with severe asthma.^233^ Although not yet in clinical practice, inhibitors of the IL‑1 pathway, such as the IL‑1 receptor antagonist, may therefore represent a future treatment option for this asthma endotype.

#### Notch signaling

Notch receptors influence T-cell differentiation and can promote a Th2-biased immune response in asthma.^234^ Inhibition of Notch signaling in animal models reduces Th2 cytokine production. Notch activity in structural cells also contributes to mucus hypersecretion and fibrotic remodeling.^235^ Although γ-secretase inhibitors can block Notch activation, their long-term use poses safety challenges due to the broad physiological roles of Notch signaling.

#### RhoA–Rho kinase (ROCK) pathway

The RhoA–ROCK pathway regulates smooth muscle contraction, cytoskeletal organization, and cell migration (Fig. 4c).^236^ In ASM, activation of RhoA and its downstream effector ROCK enhances calcium sensitivity by inhibiting myosin light chain phosphatase, leading to sustained contraction even at lower calcium levels. Upregulation of this pathway contributes to exaggerated and prolonged bronchoconstriction in asthma.^237^ ROCK signaling also supports smooth muscle proliferation and migration, key features of airway remodeling. Experimental studies have shown that ROCK inhibitors such as fasudil and Y-27632 reduce airway hyperresponsiveness and inflammation in animal models. Cytokines such as IL-13 and IL-17 may converge on RhoA–ROCK signaling to promote hypercontractility.^238^ Although ROCK inhibitors are used clinically for pulmonary hypertension, their systemic use in asthma is limited by hypotensive effects. Inhaled formulations could overcome this limitation by providing local airway relaxation while minimizing systemic exposure. Observational studies have reported that ROCK activity in airway cells correlates with disease severity.^237^

#### Calcium signaling and mitochondrial function in ASM

Airway smooth muscle cells in asthma exhibit abnormal calcium dynamics, characterized by enhanced oscillatory Ca^2+^ release in response to stimulation, leading to hypercontractility. IL-13 increases the expression of Ca^2+^ channels and ryanodine receptors in ASM, further augmenting calcium flux.^239^ Intravenous magnesium sulfate, which antagonizes calcium entry, is used clinically to promote bronchodilation during severe asthma exacerbations.^240^ Mitochondria act as calcium buffers, and mitochondrial dysfunction in asthmatic ASM—manifested as fragmentation and increased basal oxygen consumption—may amplify calcium signaling and energy demand.^241^ Restoring mitochondrial function through antioxidants or mitochondrial fusion promoters may mitigate ASM hyperresponsiveness.^242^ Metformin, an AMPK activator that improves mitochondrial efficiency, has been shown in murine models to reduce airway fibrosis and hyperreactivity.^243^ Epidemiological studies have suggested that asthmatic patients with diabetes receiving metformin experience fewer exacerbations, although confounding factors remain.^244^

#### Oxidative stress and the Nrf2 pathway

Asthmatic patients’ lungs simultaneously endure the dual oxidative stress pressure caused by inflammation and the environment. The transcription factor Nrf2 coordinates antioxidant defense by inducing genes for glutathione synthesis, superoxide dismutase, and catalase. In severe asthma and among smokers, Nrf2 responses are frequently impaired. Pharmacological activation of Nrf2, for instance, with sulforaphane analogs, may reduce oxidative injury and improve corticosteroid responsiveness, although clinical trials have produced limited and inconsistent results.^245,246^

#### Immunometabolism: mTOR and AMPK pathways

Immune cell function is tightly coupled to cellular metabolism. Effector T cells and classically activated (“M1”) macrophages favor glycolysis for rapid energy production (“Warburg effect”), whereas regulatory and quiescent cells depend on oxidative phosphorylation. In asthma, proinflammatory T cells and ILC2s often undergo metabolic reprogramming toward glycolysis. The mammalian target of rapamycin (mTOR) senses nutrient availability and promotes cell growth and Th2 differentiation. Activation of mTOR by IL-2 and TSLP enhances allergic inflammation,^247^ while pharmacologic inhibition of mTOR with rapamycin reduces airway inflammation in experimental models, although side effects preclude its long-term use. Conversely, AMPK acts as an energy sensor that shifts metabolism toward catabolic and anti-inflammatory states. As mentioned, adiponectin activates AMPK in macrophages, suppressing inflammatory mediators, and AMPK activation through metformin has been shown to promote regulatory immune phenotypes and inhibit ASM proliferation.^248^ Manipulating metabolic pathways to reprogram immune responses represents an emerging therapeutic avenue, with agents such as 2-deoxyglucose reducing Th2 cytokine production by limiting glycolysis. Such interventions may, in effect, kill two birds with one stone by acting on multiple cell types at once and thereby attenuating airway inflammation.

#### Apoptosis and autophagy pathways

Eosinophils in asthma exhibit delayed apoptosis due to survival signals provided by cytokines such as IL-5 and GM-CSF.^249^ Therapeutic agents such as benralizumab not only promote eosinophil depletion via antibody-dependent cytotoxicity (ADCC) but also interfere with these survival pathways. Autophagy regulates cytokine secretion in T cells, and genetic variations in autophagy-related genes such as ATG5 have been associated with asthma, suggesting a role in sustaining chronic inflammation.^250^

Intracellular signal transduction in asthma involves an intricate network of interacting pathways, including JAK/STAT, NF-κB, MAPK, PI3K/AKT, Notch, RhoA/ROCK, and Ca2^+^ signaling.^210^ Many established therapies act indirectly on these cascades: corticosteroids upregulate IκB and MAPK phosphatase-1 to inhibit NF-κB and MAPK activity, β_2_-agonists elevate cyclic AMP to suppress immune cell activation, and theophylline enhances HDAC and AMPK activity. Newer targeted small molecules aim to intervene more precisely, such as CRTH2 antagonists blocking prostaglandin D_2_ signaling, JAK inhibitors attenuating multiple cytokine pathways, and ROCK inhibitors relaxing ASM. Cross-talk among these pathways, exemplified by STAT6 coactivation through MAPKs and CBP/p300, introduces redundancy that may necessitate combination or upstream therapies targeting molecules such as TSLP. Achieving efficacy while minimizing off-target effects remains a major challenge. Nonetheless, a deeper understanding of these signaling networks continues to reveal new opportunities for therapeutic intervention beyond conventional receptor antagonism.

### Neural–immune crosstalk

The respiratory tract is richly innervated by both autonomic and sensory nerves. These nerves not only control the contraction and relaxation of airway muscles but also communicate closely with the immune system.^251^ This two-way conversation between nerves and immune cells, i.e., the so-called neuroimmune interaction, plays a major role in asthma.^252^ It is especially important in forms such as nonallergic hyperreactive asthma and chronic cough, where nerve activity drives much of the problem.

#### Cholinergic vs. adrenergic balance

On the efferent side, parasympathetic or cholinergic nerves release acetylcholine (ACh), which acts on muscarinic M3 receptors of the airway smooth muscle and causes the airways to narrow. In individuals with asthma, the vagus nerve is often more active than normal, making the airways more likely to constrict in response to things such as irritants or acid reflux. Therefore, drugs that block acetylcholine, such as ipratropium or tiotropium, can be very helpful, particularly for patients whose asthma shows signs of high cholinergic activity, such as baseline wheezing or vagally triggered attacks. These same nerves also stimulate mucus production from submucosal glands. Interestingly, certain immune cells called ILC2s express α7 nicotinic ACh receptors that can respond to acetylcholine as well,^253^ and when activated, these receptors actually suppress inflammatory cytokine release, suggesting that some cholinergic signals might have anti-inflammatory effects.

The sympathetic nervous system mainly affects blood vessels and glands in the lungs, while direct sympathetic control of airway muscles is limited. Instead, circulating adrenaline activates β_2_ receptors on the smooth muscle to relax the airways. Sympathetic activation therefore causes bronchodilation via β_2_ on smooth muscle and vasoconstriction via α receptors on vessels. During exercise or stress, the body’s own adrenaline helps open the airways. Medications that mimic this effect, called β_2_-agonists, are widely used for asthma relief. Beyond their muscle-relaxing effect, they may also influence the immune system by calming down immune cells such as ILC2s and DCs.^254^ Research in mice lacking β_2_ receptors shows that they develop stronger allergic inflammation, suggesting that normal adrenaline signaling usually helps keep immune reactions in check.^255^ This might also explain why overusing β_2_-agonists can sometimes worsen asthma control—by reducing receptor sensitivity and removing that natural brake on inflammation.

#### Sensory nerves

Sensory nerves in the airways, mostly C-fibers and Aδ fibers from the vagus nerve, also play a critical role in asthma symptoms.^256^ These nerves extend throughout the airway epithelium and smooth muscle, where they serve as the main sensors of physical and chemical stimuli. Each sensory nerve ending contains specialized receptors known as transient receptor potential (TRP) channels, particularly TRPV1 and TRPA1.^257^ TRPV1 responds to heat and capsaicin—the compound that gives chili peppers their burn—while TRPA1 is activated by chemical irritants such as acrolein, components of cigarette smoke, cold air, and oxidative stress. When these receptors are stimulated, they generate electrical impulses that travel to the brain, triggering reflex bronchoconstriction and coughing. At the same time, they trigger a local axon reflex, which causes the nerve endings themselves to release signaling molecules known as neuropeptides, including substance P, neurokinin A, and calcitonin gene-related peptide (CGRP). These neuropeptides initiate what is known as neurogenic inflammation. Substance P and neurokinin A promote airway constriction, increase vascular leakage, and stimulate mucus secretion by activating neurokinin receptors on airway cells. CGRP, in contrast, mainly causes blood vessels to dilate. Together, these effects contribute to the airway hyperreactivity and inflammation characteristic of asthma.

People with asthma often have hypersensitive sensory nerves. For instance, when they inhale capsaicin, a substance that activates TRPV1, they tend to cough more readily and intensely than healthy individuals, reflecting an exaggerated TRPV1 response. Similarly, breathing cold air or irritants such as chlorine can trigger bronchospasm in asthmatic patients, implicating heightened TRPA1 activity. Animal studies provide strong support for these mechanisms. Experiments show that blocking TRPA1 can markedly reduce the signs of allergen-induced asthma, including inflammation and airway overreactivity.^258^ TRPA1 is not limited to nerves—it has also been detected on certain immune cells, such as specific CD4^+^ T-cell subsets,^259^ suggesting that it might influence inflammation directly through immune signaling as well.

Because of these findings, researchers are exploring TRP channel antagonists as potential treatments for asthma and chronic cough.^260^ In animal models, TRPA1 blockers have been shown to reduce both cough and airway inflammation.^261^ However, none of these drugs have yet entered human trials. One concern is that completely blocking TRPA1 might dull protective sensations, such as the cough reflex, which could increase the risk of aspiration.

#### NMU–ILC2 axis

Another fascinating connection between the nervous and immune systems involves a neuropeptide called neuromedin U, or NMU.^262,263^ This molecule is released by certain cholinergic sensory nerves in the lungs and can strongly activate ILC2s through their NMUR1 receptors.^264,265^ NMU binding to ILC2s causes an immediate burst of IL-5 and IL-13 release from ILC2s, even more rapidly than cytokine-driven activation.^187^ This provides a mechanism for the ultrafast eosinophil recruitment observed in some allergen challenges within 1–2 h, occurring before Th2 cells can respond.^262^ Studies show that blocking NMU or its receptor reduces asthma inflammation, while giving NMU alone can trigger asthma-like eosinophilia even in the absence of allergens. This means that overactive nerves can directly drive inflammation, helping explain why some people develop “intrinsic” asthma without allergic triggers.^266^ It also helps clarify why treatments that block upstream immune signals, such as TSLP inhibitors, can work even for nonallergic asthma—they may indirectly modulate this nerve–immune activation.

#### Cough-variant asthma and neural sensitization

Some patients with asthma mainly experience chronic cough rather than wheezing or shortness of breath. This form, known as cough-variant asthma, is characterized by an exaggerated cough reflex to everyday triggers such as irritants, cold air, or even talking. It overlaps with cough hypersensitivity syndrome, in which TRPV1- and TRPA1-expressing sensory nerves become overly excitable.^267^ Inflammatory molecules such as prostaglandins, bradykinin, and IL-13 can make cough-sensitive nerves more reactive by increasing TRP channel expression. At the same time, persistent nerve activation keeps inflammation going by releasing more neuropeptides. This insight has led to new treatment ideas that target nerve activity. For example, P2X3 antagonists, which block ATP-sensitive channels on vagal cough fibers, are currently being tested for refractory chronic cough and might also benefit those with asthma-related cough.^268,269^

#### Stress and emotions

Psychological stress can further amplify this neuroimmune network. Stress tends to activate both the vagus tone and the sympathetic system and can trigger the release of neuropeptides such as substance P in the brain. Many people with asthma notice that their symptoms worsen during periods of stress or anxiety.^270^ This may be because stress enhances cholinergic reflexes and increases the release of inflammatory neuropeptides, making the airways more reactive. The so-called “psychogenic asthma” still has physiologic underpinnings in neural pathways.

#### Therapeutic implications

Recognizing the neural component in asthma has opened new therapeutic possibilities. Bronchial thermoplasty, which uses controlled radiofrequency energy to reduce airway smooth muscle, may also affect nearby airway nerves. Post-thermoplasty patients have sometimes reported reduced cough and improved symptoms beyond what is expected from ASM reduction alone, raising speculation that nerve modulation may be part of its effect.^271^ Future treatments may further explore this neurogenic dimension. Potential strategies include blocking TRP channels such as TRPA1 or TRPV1, inhibiting NK-1 receptors to counteract substance P activity,^272^ or more broadly modulating cholinergic signaling beyond current long-acting muscarinic antagonists. Although an NK-1 blocker tested for asthma showed limited efficacy,^273^ likely due to overlapping pathways, these targets remain of interest.^274^ Tiotropium, already recommended for moderate-to-severe asthma, reflects the clinical importance of vagal tone control. Looking ahead, a selective TRPA1 inhibitor that can be safely delivered to the lungs could offer dual benefits by reducing both neurogenic inflammation and cough, an appealing prospect if future studies confirm its safety. As we broaden asthma treatment beyond purely immune suppression, addressing neural contributors holds promise for those with hard-to-treat symptoms such as chronic cough or exercise-induced bronchospasm that are less steroid-responsive.

### Endotypes, phenotypes, and treatable traits

**Fig. 7:**
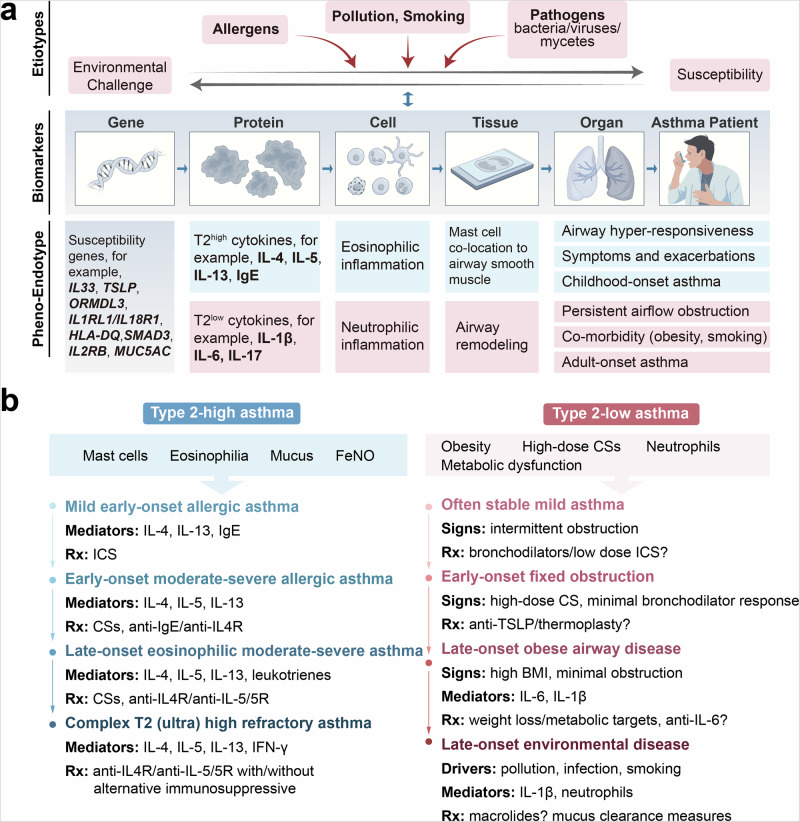
Integrated framework of asthma phenotypes, endotypes, and biomarkers. **a** Multiscale immunopathology of asthma linking genetic susceptibility and environmental exposure to immune polarization and tissue remodeling. T2-high disease is driven by epithelial alarmins (TSLP, IL-33, IL-25) and downstream IL-4/IL-5/IL-13 signaling involving Th2 cells, ILC2s, eosinophils, and mast cells, whereas T2-low endotypes feature neutrophilic or paucigranulocytic inflammation mediated by IL-17, IL-6, IFN-γ, and TNF-α. Representative biomarkers include gene loci (*ORMDL3*, *IL33*, *IL1RL1*), serum periostin, sputum cytology, and exhaled NO. **b** Clinical and therapeutic continuum of T2-high and T2-low asthma. The T2-high phenotype ranges from mild, corticosteroid-responsive early-onset allergic asthma to severe late-onset eosinophilic asthma or complex (ultrahigh) T2-high asthma, requiring biologic treatments such as anti-IL-5, anti-IL-4Rα, or anti-TSLP. The T2-low phenotype includes noneosinophilic, neutrophil-predominant, and obesity-associated subtypes, which are less responsive to steroids but may benefit from macrolides, bronchial thermoplasty, and emerging cytokine or kinase inhibitors. CS, corticosteroid; FeNO, fractional exhaled nitric oxide; Rx, treatments. This figure was manually produced by the author(s) using Adobe Illustrator, with no assistance from generative AI. Figure 7a was modified from our previous work (Xie et al., 2025, Respir. Res.).^430^

Asthma is increasingly recognized not as a single disease entity but as a heterogeneous syndrome encompassing multiple phenotypes and underlying endotypes.^275^ Over the past decade, a paradigm shift has occurred from a purely clinical classification to one based on identifiable phenotypic clusters and distinct molecular endotypes (Fig. 7a). This approach aims to enable precision therapy by targeting the specific disease mechanisms present in each individual, a concept referred to as “treatable traits.”^276^

#### T2-high and T2-low endotypes

Broadly, asthma endotypes are often categorized according to the presence or absence of type 2 (T2) inflammation (Table 1 and Fig. 7b). The T2-high endotype is characterized by eosinophilic airway inflammation and elevated levels of type 2 cytokines such as IL-4, IL-5, and IL-13.^277,278^ Typical features include increased fractional exhaled nitric oxide (FeNO), blood eosinophilia, and evidence of atopy or allergic sensitization. Clinically, these patients often have childhood-onset disease, allergic comorbidities, and a favorable response to corticosteroids and T2-targeted biologics. T2-high asthma encompasses both classic allergic asthma and late-onset eosinophilic asthma, the latter being nonallergic but IL-5-driven. Common biomarkers include FeNO, blood eosinophil count, serum IgE, periostin, and sputum eosinophils. These markers predict response to therapy; for instance, elevated FeNO or eosinophil levels are associated with better outcomes following ICS or anti-IL-5 therapy.

**Table 1:** Asthma endotypes from research to practice

		Type 2-“high” asthma	Type 2-“low” asthma
Lab	Epithelial cells	secreted TSLP, IL-33, and IL-25	secreted IL-1β and IL-23
	DCs	DC2 express IL-4, OX-40 L, CCL17, and PGE2	DCs secreted IL-6, IL-23 and TGF-β
	T_H_ cells	T_H_2 secreted IL-4, IL-5, IL-13, and IL-31 T_H_9 secreted IL-9	T_H_1 secreted IFN-γ and TNF-α T_H_17 secreted IL-17
	ILCs	ILC2 secreted type 2 cytokines	ILC1s secreted IFN-γ and TNF-α ILC3s secreted IL-17
	NKT cells	NKT cells secreted type 2 cytokines	Monocyte and NKT cells secreted IL-8
	B cells	IgE class-switched B cells	—
	Mast cells	Mast cells secreted proteas and PGD2	—
	Effector cells	Eosinophils secreted IL-4, IL-5, IL-13, granule proteins (EDN, ECP, EPO, MBP), and cys-LTs	Neutrophilic or paucigranulocytic
Clinic	Age of onset	Earlier onset (children)	Later onset (adult)
	Triggers	Allergen, pathogen infection; comorbid CRSwNP	Air pollutants, smoking
	Clinical behavior	Often associated with allergic rhinitis, positive skin prick test to aeroallergens or presence of allergen-specific IgE	Corticosteroid resistant, absent of eosinophilia
	Endotyping indicators	Blood eosinophil counts **≥ 150/μL**, and/or FeNO **≥ 20ppb**, and/or sputum eosinophils **≥ 2%**, and/or asthma is clinically allergen-driven [GINA 2024]	—
	Biomarkers	Blood/sputum eosinophils, serum IgE, FeNO, serum periostin	—
	Inflammatory mediators	IgE, IL-5, IL-13, IL-4	IL-17, IL-1β, IL-6, TNF-α
	Obesity/metabolic dysfunction	May be present	Often present
	Lung function	More obstruction	Less obstruction
	FeNO	Normal to elevated	Low to normal
	Life-threatening exacerbations	More exacerbations	Fewer exacerbations
	Medication sensitivity	More responsive to corticosteroids and bronchodilators	Less responsive to corticosteroids and bronchodilators

In contrast, T2-low asthma lacks the typical T2 inflammatory signature.^8^ Airway inflammation is often neutrophilic or pauci-granulocytic, with mechanistic pathways involving Th1 (IFN-γ-driven), Th17 (IL-17-mediated), or intrinsic innate immune activation.^279^ Patients with T2-low disease are typically older, may have obesity or a history of smoking, and exhibit poor responsiveness to ICS. FeNO and IgE levels are usually normal, and symptoms often persist due to airway remodeling or non-eosinophilic mechanisms. Currently, no approved biologics specifically target the T2-low endotype. However, macrolide antibiotics have demonstrated benefits in some cases. In the AMAZES trial, azithromycin significantly reduced exacerbation frequency, particularly in patients with non-eosinophilic asthma, suggesting a potential benefit through neutrophil modulation and antimicrobial effects.^101^ Additional approaches include bronchial thermoplasty and novel agents targeting neutrophil recruitment pathways, such as CXCR2 antagonists.^280^

#### Biomarkers

FeNO is generated by inducible nitric oxide synthase (iNOS) in airway epithelial cells in response to IL-13.^281^ Elevated FeNO levels, commonly defined as >25 ppb in adults and >20 ppb in children, with higher cutoffs such as 35–50 ppb often used for greater specificity, indicate ongoing type 2 inflammation and correlate with airway eosinophilia. FeNO is particularly useful for identifying patients likely to benefit from ICS or IL-4/IL-13–targeted therapies such as dupilumab, which produces marked and rapid reductions in FeNO.^282,283^ However, FeNO levels are affected by comorbid allergic rhinitis, smoking, and current ICS use and should therefore be interpreted in the clinical context. Despite these limitations, FeNO remains a convenient, noninvasive biomarker for assessing airway inflammation, treatment adherence, and therapeutic response. Importantly, recent patient-level meta-analyses from the ORACE and ORACE2 consortia demonstrate that elevated FeNO (together with blood eosinophils) identifies asthmatic patients at increased risk of future severe attacks, suggesting a role not only in monitoring but also in risk stratification and preventive strategy selection.^284,285^

Blood eosinophil count is a practical surrogate for type 2 airway inflammation.^286^ Thresholds of ≥150–300 cells/µL are commonly used to identify eosinophilic asthma and to predict therapeutic response to anti–IL-5 and anti–IL-4R biologics.^287,288^ Although its correlation with sputum eosinophilia is imperfect, peripheral eosinophil measurement is widely accessible and forms an essential component of severe asthma evaluation.^289^ Markedly elevated eosinophil counts (e.g., >1000 cells/μL) should prompt consideration of eosinophilic syndromes such as eosinophilic granulomatosis with polyangiitis (EGPA).^290^

Induced sputum eosinophils provide the most direct and accurate measure of the airway inflammatory phenotype.^291^ A threshold of ≥2–3% eosinophils is generally accepted to define eosinophilic asthma.^292^ Studies using sputum-guided management strategies have demonstrated reduced exacerbation rates by maintaining eosinophil levels below this threshold.^291,292^ However, sputum induction requires specialized expertise and is not routinely available in most clinical settings.

Elevated serum total IgE is a hallmark of allergic asthma and historically guided eligibility for anti-IgE therapy (omalizumab), which required both allergen sensitization and IgE levels within a defined dosing range. Allergen testing via serum-specific IgE or skin prick testing helps identify relevant triggers.^293^ Very high total IgE levels may raise suspicion for conditions such as allergic bronchopulmonary aspergillosis (ABPA).

Periostin, an extracellular matrix protein induced by IL-13 in epithelial cells and fibroblasts, reflects type 2–driven airway remodeling.^294^ Although early studies suggested that elevated periostin predicted an enhanced response to IL-13 blockade, substantial interindividual variability has limited its clinical utility,^295^ and it remains primarily a research biomarker.

Breathomics, the analysis of exhaled volatile organic compounds, represents an emerging approach to noninvasive asthma phenotyping. Specific VOC patterns can distinguish eosinophilic from neutrophilic asthma,^296^ and electronic-nose technologies are being developed for clinical application, potentially enhanced by AI-based pattern recognition.

Several additional biomarkers have been proposed. These include YKL-40 (chitinase-3-like-1), which is elevated in subsets of asthma and associated with neutrophil/macrophage activation;^297^ dipeptidyl-peptidase-4 (DPP4/CD26), which is upregulated by IL-13 and may serve as a marker of IL-13 pathway activity;^298^ club cell 16-kDa secretory protein (CC16), which is typically reduced in asthma and reflective of epithelial integrity;^299,300^ and resistin-like molecule-α (RELM-α), an IL-13–induced mediator linked to mucus hypersecretion.^301^

#### Clinical phenotypes

Large cohort analyses, such as those from U-BIOPRED and SARP,^302,303^ have identified a spectrum of clinical phenotypes beyond the simple T2-high/T2-low classification (Table 2).

**Table 2:** Phenotypes and endotypes of asthma

Endotype	Phenotype	Biomarkers	Clinical characteristics
T2-high	Atopic (early-onset)	Blood/sputum eosinophil count; allergic sensitization; high total IgE; high FeNO; periostin; DDP4, plasma galectin-3	Early onset, steroid responsive, often comorbid allergic rhinitis
	Late-onset	Blood/sputum eosinophil count; high FeNO; periostin; DPP4; plasma galectin-3	Steroid refractory, may have CRSwNP, responds well to anti-IL-5/IL-4R
	AERD	Blood/sputum eosinophil count; urinary LTE4; periostin; DPP4; plasma galectin-3	Adult onset, triad of asthma, severe type 2 inflammation
T2-low	Non-Atopic	Sputum neutrophil count	Adult onset, ICS-poor response
	Smokers	Sputum neutrophil count	Older adults, asthma with COPD-like physiology, fixed obstruction, ICS-resistant
	Obesity-related	↑ Serum IL-6; ↓ adiponectin, ↑ leptin	Female predominant, low FeNO
	Very late onset	Sputum neutrophil count	>50–65 at onset, frequent comorbidities, airway remodeling prominent

#### Allergic, early-onset asthma

Typically, begins in childhood or adolescence, associated with eczema, allergic rhinitis, and allergen sensitization. It is generally T2-high and of moderate severity, although some cases progress to severe disease.

#### Late-onset eosinophilic asthma

Characterized by adult onset, high eosinophil counts, and frequent comorbid sinusitis or nasal polyps, as seen in aspirin-exacerbated respiratory disease. Although not clearly allergic, this phenotype responds well to IL-5- or IL-4Rα-targeted biologics.

#### Obesity-related asthma

More common in adult women and linked to metabolic dysfunction.^304^ Symptoms of dyspnea may be disproportionate to the degree of lung function impairment. Affected individuals often exhibit low-grade neutrophilic inflammation or none (“paucigranulocytic”). The mechanical effects of an enlarged thoracic cavity and abdomen reduce lung volumes and increase airway closure.^305^ Systemic inflammation originating from adipose tissue (e.g., IL-6 and leptin signaling) can blunt steroid responsiveness and promote a Th1/Th17 environment.^306^ Weight loss via diet or bariatric surgery has been shown to improve asthma control and lung function in these patients, highlighting the phenotype.^307^ These patients often have low FeNO and not high IgE. Biologics help if they do have high eosinophil counts, but many are T2-low and mainly need weight management and possibly macrolides.

#### Exercise-induced bronchoconstriction (EIB)

EIB is characterized by transient airflow limitation triggered by exercise, largely mediated by airway dehydration and cooling that stimulate mast cell activation.^308^ It commonly affects younger individuals with mild or no persistent asthma. Although defined by its trigger rather than inflammatory profile, type 2 inflammation is often present. Pretreatment with bronchodilators or mast cell stabilizers is beneficial, and EIB commonly overlaps with allergic asthma.

**Cough-variant asthma** presents mainly with chronic cough and minimal wheezing, potentially representing an early or milder disease form or reflecting airway neural hypersensitivity.^309,310^

#### Asthma with fixed airflow limitation

Long-standing disease leading to structural remodeling and persistent obstruction (nonreversible FEV_1_).^311^ This phenotype overlaps with COPD functionally and has limited reversibility; interventions targeting remodeling (e.g., anti-IL-13 or anti-TGF-β strategies) remain experimental.^312^

#### Smoking-associated asthma

Shares features with COPD and is characterized by neutrophilic inflammation, corticosteroid resistance, and oxidative stress, consistent with a T2-low profile.

#### Treatable traits approach

**Fig. 8:**
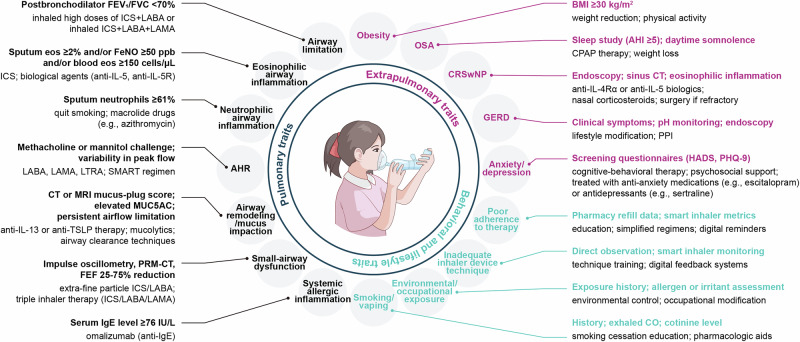
Treatable traits in asthma, including pulmonary, extrapulmonary, and behavioral/lifestyle factors. Pulmonary traits include airway inflammation patterns, small airway dysfunction, and mucus plugging. Extrapulmonary traits include obesity, nasal polyps, chronic rhinosinusitis, gastroesophageal reflux, and mental health comorbidities. Behavioral and lifestyle traits cover smoking, medication adherence, and environmental or occupational exposures. Identifying and addressing these treatable traits is essential for individualized asthma management. BMI body-mass index, GERD gastroesophageal reflux disease, OSA obstructive sleep apnea, AHR airway hyperreactivity, CRSwNP chronic rhinosinusitis with nasal polyps, AHI apnea-hypopnea index, CPAP continuous positive airway pressure, PPI proton pump inhibitor. The author(s) hand-drew this figure using Adobe Illustrator

Given this heterogeneity, the framework of “treatable traits” has been proposed to guide individualized management. Treatable traits are clinically or biologically defined characteristics that can be identified and specifically targeted in each patient.^276,313^ These may be pulmonary (e.g., eosinophilic inflammation, airway hyperreactivity, small-airway dysfunction), extrapulmonary (e.g., obesity, chronic rhinosinusitis, anxiety), or behavioral (e.g., poor adherence, incorrect inhaler technique) (Fig. 8). Multiple traits may coexist and should be addressed concurrently.

For severe asthma, some key treatable traits and corresponding interventions include the following:

#### Eosinophilic inflammation

Managed with high-dose ICS or biologics targeting IL-5/IL-5R or IL-4Rα, aiming to reduce sputum eosinophils below 3%.

#### Allergic sensitization

Addressed with omalizumab and allergen avoidance or immunotherapy when feasible.

#### Frequent exacerbations

Managed according to the underlying driver—T2 inflammation with biologics, infection-related exacerbations with macrolides, and adherence or inhaler issues with education.

#### Mucus plugging

May benefit from hydration, mucolytics, and biologics such as tezepelumab, which has been shown to reduce mucus impaction.

#### Small-airway dysfunction

Identified by oscillometry or CT evidence of air trapping; treatable with extrafine particle ICS or triple therapy (ICS/LABA/LAMA) to improve distal airway deposition and lung emptying.^314^

#### High cholinergic tone

Improved with long-acting muscarinic antagonists such as tiotropium.

#### Neutrophilic inflammation

May respond to macrolides and, potentially, to biologics targeting upstream alarmins such as TSLP.

#### Exercise-induced symptoms

Managed with preexercise β_2_-agonists or leukotriene receptor antagonists such as montelukast.

#### Psychological dysfunction

Anxiety and panic can mimic or worsen asthma symptoms and warrant psychological assessment and intervention.

In essence, while we define broad endotypes and phenotypes, each asthma patient is unique. Identifying their specific pathogenic factors and biomarkers is key to optimizing therapy. Thanks to biomarkers such as FeNO, blood eosinophil counts, and IgE levels, we can relatively easily classify many patients as either T2-high or T2-low and select treatment regimens accordingly. Notably, phenotypes can shift over time or with age. A child with allergic asthma may grow into an adult whose asthma becomes more eosinophilic with less allergy, or could develop fixed obstruction decades later. Therefore, it is crucial to periodically reassess phenotypes and traits. Indicators measured at clinic visits, such as blood eosinophil counts and FeNO, can signal whether inflammation is off-target or if a previously T2-low patient has shifted to T2-high due to certain changes—such as discontinuation of ICS or exposure to new allergens—and vice versa. The vision of precision medicine in asthma involves multidimensional assessment of each patient, addressing all relevant treatable traits to minimize disease activity and improve quality of life.

Finally, the refinement of asthma endotyping aims to achieve the optimal therapeutic outcome—disease remission.^315^ With the advent of effective targeted therapies and comprehensive management of comorbid traits, some patients can attain a sustained state of “clinical remission”, characterized by the absence of symptoms and exacerbations and preservation of normal lung function for at least 12 months while continuing treatment. In certain cases, “complete remission” without ongoing therapy may even be achievable. Recent consensus statements and clinical trials have begun to adopt remission as a key therapeutic endpoint, representing a paradigm shift from the traditional view of asthma as a lifelong, chronically “controlled” condition.^316^

## Therapeutic and translational strategies

Effective management of asthma requires a comprehensive approach that simultaneously controls chronic airway inflammation, alleviates bronchoconstriction, and reduces exposure to modifiable risk factors or triggers. Asthma therapy has evolved from a “one size fits all” approach of bronchodilators and corticosteroids to a stratified model where specific treatments target distinct pathways relevant to an individual’s asthma endotype. This section covers the spectrum of treatments, linking how mechanistic insights have informed these strategies.

### Conventional pharmacotherapy

**Fig. 9:**
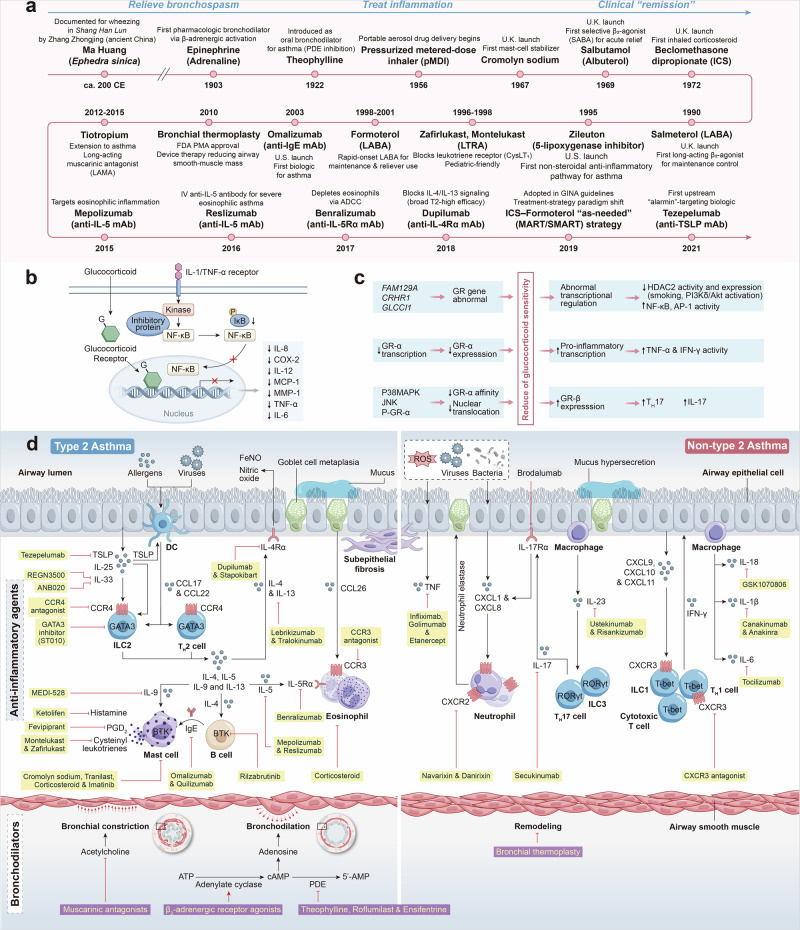
Therapeutic landscape and mechanistic basis of asthma treatment. **a** The trajectory of asthma therapy has shifted from traditional symptom-based management toward mechanism-driven precision medicine, reflecting the ongoing translation of fundamental discoveries into clinical innovation. **b** Mechanisms of glucocorticoid action. Glucocorticoids exert their anti-inflammatory effects by activating cytoplasmic glucocorticoid receptors that translocate to the nucleus, repress NF-κB and AP-1 signaling, and induce anti-inflammatory genes such as IL-10 and MKP-1 through HDAC2-dependent chromatin remodeling. **c** Mechanisms of corticosteroid resistance. Impaired GR expression or phosphorylation, oxidative/nitrative stress–induced HDAC2 loss, p38 MAPK activation, and PI3K/AKT pathway upregulation contribute to persistent inflammation and reduced steroid sensitivity. **d** Integrated therapeutic strategies targeting airway inflammation and hyperresponsiveness. Current asthma therapy is built on two major pillars. One focuses on suppressing inflammation through small-molecule drugs or investigational biologics that modulate cytokine, chemokine, or alarmin signaling to reduce immune activation. The other focuses on bronchial relaxation, including receptor-mediated and interventional treatments, to restore airway patency and function. This figure was originally hand-drawn by the author(s) using Adobe Illustrator

The foundation of asthma management has long relied on inhaled pharmacotherapy designed to suppress chronic airway inflammation and alleviate bronchoconstriction. These conventional treatments are generally divided into two main categories: controller medications, which are taken regularly to maintain long-term disease stability, and reliever medications, which are used as needed to rapidly relieve acute symptoms (Fig. 9a).

#### Inhaled corticosteroids (ICS)

Introduced in the 1970s, ICS remains the cornerstone of asthma control for persistent disease.^317^ These agents, such as beclomethasone, budesonide, fluticasone, mometasone, and ciclesonide, exert their effects by entering airway epithelial and immune cells, where they bind to glucocorticoid receptors. The activated receptor complex then translocates to the nucleus and modulates gene transcription, enhancing the expression of anti-inflammatory mediators such as IL-10 and IL-1RA while suppressing proinflammatory signaling pathways through inhibition of transcription factors, including NF-κB and AP-1 (Fig. 9b). The recruitment of HDAC2 further contributes to the suppression of inflammatory gene expression.^65^ The overall result of these molecular actions is a marked reduction in the production of cytokines (IL-5, IL-13, IL-17, etc.), chemokines, and eicosanoids, accompanied by improved responsiveness to β_2_-agonists. Clinically, ICSs alleviate symptoms, enhance lung function, and reduce the frequency of exacerbations by approximately half or more in patients with eosinophilic asthma. They also gradually attenuate airway hyperresponsiveness and lower eosinophil counts in airway tissue and sputum, promoting epithelial repair, although some structural remodeling, such as fixed basement membrane thickening, may persist.

Despite their proven efficacy, ICSs are not universally effective. A subset of patients, particularly those with neutrophilic or severe asthma, exhibit steroid resistance due to factors such as oxidative stress–induced loss of HDAC2 activity or overexpression of multidrug resistance proteins in immune cells (Fig. 9c).^318^ Long-term or high-dose use can also produce adverse effects, including oral candidiasis, dysphonia, and systemic complications, such as adrenal suppression, osteoporosis, or cataract formation. Current treatment guidelines therefore emphasize the importance of using the lowest effective dose and incorporating additional controller therapies rather than simply ramping up ICS endlessly in severe cases. Nevertheless, for the majority of patients, ICSs have been transformative, substantially reducing asthma-related hospitalizations and mortality globally.

#### β2-adrenergic agonists

These bronchodilators stimulate β_2_ receptors on airway smooth muscle, activating adenylate cyclase, raising cAMP, and causing relaxation and mast cell stabilization. Short-acting β_2_ agonists (SABA), such as albuterol (salbutamol), provide quick relief of acute bronchospasm and are commonly referred to as “rescue” medications. Their onset of action occurs within minutes, reaches a peak by 15 min, and lasts for 4 to 6 h. Historically, these agents were used as needed and even prophylactically before exercise. However, reliance on SABA alone can mask worsening airway inflammation and has been linked to an increased risk of exacerbations. Frequent SABA use, particularly exceeding one inhaler per month, has been associated with higher mortality,^11^ possibly due to cardiac side effects or receptor downregulation. For this reason, current clinical guidelines no longer recommend SABA monotherapy. Even patients with mild asthma are now advised to receive ICS to address the underlying inflammatory component of the disease.

Long-acting β_2_ agonists (LABAs), including formoterol, salmeterol, vilanterol, and olodaterol, have a prolonged bronchodilatory duration of approximately 12–24 h. They are used for maintenance therapy in moderate to severe asthma but should always be prescribed in combination with ICS. LABA monotherapy is contraindicated because earlier studies in the 1990s demonstrated an increased risk of asthma-related death when inflammation was not simultaneously controlled with corticosteroids. The combination of ICS and LABA produces a synergistic effect: corticosteroids upregulate β_2_ receptor expression and prevent receptor desensitization, while long-acting agonists enhance corticosteroid receptor activation and nuclear translocation. This combination improves symptom control and lung function and reduces exacerbation risk. Commonly used formulations include budesonide–formoterol, fluticasone–salmeterol, fluticasone–vilanterol, beclomethasone–formoterol, and mometasone–formoterol.

Formoterol is particularly notable because it has a rapid onset of action, comparable to short-acting agents, while providing long-lasting bronchodilation.^319^ This pharmacological profile allows it to serve both as a maintenance and as a reliever medication. The single-maintenance-and-reliever therapy (SMART) regimen, which employs an ICS–formoterol combination for both regular use and symptom relief, has demonstrated substantial clinical benefits.^320^ Large clinical trials such as SYGMA have shown that as-needed budesonide–formoterol therapy reduces severe exacerbations by more than 60% compared with SABA use alone in patients with mild asthma, without compromising symptom control.^321^ In moderate to severe asthma, maintenance and reliever therapy (MART) with a single inhaler containing budesonide–formoterol or beclomethasone–formoterol results in approximately 30% fewer exacerbations than fixed-dose ICS/LABA therapy combined with a separate SABA reliever.^322^ Importantly, this strategy relies on the rapid onset of formoterol, which makes it suitable for both maintenance and rescue use, an advantage not shared by salmeterol, whose slower onset of 10–20 min precludes its use for immediate symptom relief.^323^

#### Anticholinergics

Anticholinergic bronchodilators, such as ipratropium and tiotropium, act by blocking M_3_ muscarinic receptors on airway smooth muscle, thereby reducing vagally mediated bronchoconstriction. Ipratropium, a short-acting muscarinic antagonist (SAMA), has a relatively slow onset of action of approximately 15 min and produces less potent bronchodilation than SABA. For this reason, it is generally not used as a first-line agent for rapid symptom relief, except in patients who cannot tolerate SABA, such as those with certain cardiac arrhythmias, or in individuals who experience tremor as a side effect of β_2_ agonist therapy.

Tiotropium, a long-acting muscarinic antagonist (LAMA), has proven to be a valuable add-on treatment for patients with moderate-to-severe asthma who remain symptomatic despite the use of ICS/LABA. It has been shown to improve lung function and reduce the frequency of exacerbations in this population. More recently, single inhaler formulations of ICS/LABA/LAMA (e.g., fluticasone furoate [FF] + umeclidinium [UMEC] + vilanterol [VI]; beclomethasone dipropionate [BDP] + formoterol fumarate [FOR] + glycopyrronium bromide [GLY]) have become available, offering a convenient option for patients with difficult-to-control asthma and improving adherence by simplifying treatment regimens.^324^

Clinical trials such as CAPTAIN for FF/UMEC/VI^325^ and TRIMARAN/TRIGGER for BDP/FOR/GLY^326^ have shown that triple therapy can further improve lung function and modestly reduce exacerbations compared with ICS/LABA. The benefit appears greatest in patients with frequent symptoms or a history of exacerbations. Triple combinations are now recommended as an option for Step 5 therapy, particularly in those requiring frequent bronchodilator use or with features of COPD overlap. A network meta-analysis indicated that high-dose ICS/LABA/LAMA provides better protection against exacerbations than medium-dose regimens or ICS/LABA alone.^327^ The effect seems stronger in patients with eosinophilic inflammation. Notably, the reduction in exacerbations was greater in individuals with blood eosinophil counts above 300 cells per microliter than in those below that threshold, suggesting that type 2-high severe asthma may derive the greatest benefit from triple therapy.

#### Leukotriene receptor antagonists (LTRAs)

Montelukast and zafirlukast are LTRAs that act by blocking the cysteinyl leukotriene type 1 (CysLT_1_) receptor. Through this mechanism, they inhibit the biological actions of leukotrienes C4, D4, and E4, which are potent bronchoconstrictors and proinflammatory mediators primarily released by eosinophils and mast cells. These agents are administered orally, typically once daily, and exhibit moderate efficacy compared with ICS. LTRAs can serve as add-on therapy for patients with mild to moderate asthma or as alternative options for those who are unable or unwilling to use ICSs, although ICSs generally provide superior control of airway inflammation. They are especially beneficial in specific asthma phenotypes, such as AERD and exercise-induced bronchoconstriction, conditions in which leukotriene pathways play a central role. When taken before physical activity, LTRAs can effectively reduce exercise-induced asthma. Montelukast has the additional advantage of improving allergic rhinitis symptoms, making it especially useful for patients who present with both asthma and allergic rhinitis.

In terms of safety, LTRAs are generally well tolerated. However, rare neuropsychiatric adverse events, including mood alterations, vivid dreams, and, in a few cases, suicidal ideation, have been reported, prompting a warning from the U.S. Food and Drug Administration. Hepatotoxicity is another potential concern, more commonly associated with zafirlukast, whereas montelukast has a more favorable hepatic safety profile. In pediatric populations, montelukast is frequently prescribed for mild asthma or for children with concomitant allergic rhinitis. Another agent within this class, zileuton, acts as a 5-lipoxygenase inhibitor that suppresses leukotriene synthesis directly. Despite its mechanistic distinction, zileuton is less frequently used in clinical practice due to the need for regular monitoring of liver function and the associated risk of hepatotoxicity.

#### Theophylline and PDE inhibitors

Methylxanthines such as theophylline were once mainstay oral bronchodilators in asthma and COPD management. Their primary mechanism involves inhibition of phosphodiesterase, leading to elevated intracellular cAMP levels, a process reminiscent of β-agonist activity. Beyond bronchodilation, theophylline exerts mild anti-inflammatory effects, possibly via HDAC2 activation and some IL-10 induction. Despite these properties, its clinical use has markedly declined. The drug’s narrow therapeutic window and the potential for serious side effects such as arrhythmias and seizures have relegated it to a third-line option.

In recent years, low-dose theophylline has drawn attention for its potential to restore HDAC2 activity in steroid-resistant asthma, although this approach remains largely experimental. Moderate doses can offer modest bronchodilation and may enhance corticosteroid responsiveness, but the requirement for blood level monitoring makes routine use impractical. Consequently, it is mostly obsolete in countries with access to more modern agents but is still used in some developing regions for cost reasons.

A more recent development in this pharmacologic class involves selective phosphodiesterase-4 (PDE4) inhibitors, such as roflumilast, which are approved for use in chronic obstructive pulmonary disease (COPD). By suppressing cytokine release from inflammatory cells, these agents provide anti-inflammatory benefits that extend beyond bronchodilation. Clinical trials in asthma populations have demonstrated modest improvements in lung function, yet gastrointestinal side effects, including nausea and weight loss, have limited their acceptance. Currently, an inhaled dual PDE3/4 inhibitor, ensifentrine, is under investigation. This compound may offer a balanced combination of bronchodilation and anti-inflammatory effects, potentially with a more favorable tolerability profile than earlier agents.

#### Oral corticosteroids (OCS)

Prednisone, prednisolone and other systemic corticosteroids remain highly effective in controlling severe asthma exacerbations as well as chronic, difficult-to-treat asthma. A short “burst” course of 5–10 days often restores stability and prevents deterioration. However, their systemic toxicity makes them unsuitable for long-term use. Prolonged therapy can cause osteoporosis, diabetes, hypertension, cataracts, adrenal suppression, weight gain, and infections.

Because of these risks, OCS are now reserved for patients with severe disease that cannot be managed by other treatments. Modern practice focuses on reducing steroid exposure through alternative therapies such as biologics. Trials including SIRIUS,^328^ ZONDA,^329^ PONENTE,^330^ VENTURE^331^, and WAYFINDER^332^ have shown that biologics can markedly lower the need for daily corticosteroids. Many patients have been able to stop prednisone completely or maintain control with minimal doses. Achieving an “OCS-free remission” is now a key goal in severe asthma management, as it lessens both disease burden and treatment-related harm.

#### Cromones

Cromolyn sodium and nedocromil are mast cell stabilizers once used for mild asthma and exercise-induced symptoms, especially in children. They block mast cell degranulation and slightly dampen airway nerve activity. Although very safe, their limited potency and need for frequent dosing made them impractical. With the rise of ICS, they have largely fallen out of use, although cromolyn is still occasionally given by nebulization in young children or before exercise.

#### Allergen immunotherapy (AIT)

For patients with allergic asthma, especially if mild and driven by a single allergen such as dust mites or pollens, subcutaneous or sublingual immunotherapy can be considered. AIT involves repeated exposure to small amounts of the allergen to promote immune tolerance.^333^ This process is marked by increased production of allergen-specific IgG4, the so-called blocking antibody, and by the induction of Tregs and IL-10.^334,335^ Over 3–5 years, AIT can reduce asthma symptoms and medication needs and may have sustained benefits after completion. However, AIT carries a risk of allergic reactions, so patient selection and concomitant ICS use are important.^336^ The first FDA-approved sublingual immunotherapy tablets for dust mite and grass allergies have also been shown to reduce asthma symptoms in patients with allergic rhinitis, suggesting possible preventive value. Other experimental forms of “immune therapy,” such as probiotics or helminth exposure designed to modulate immune responses, remain under investigation. Although conceptually appealing as a way to mimic the protective “farm effect,” their results thus far have been inconsistent and have not yet influenced clinical practice.

#### Bronchial thermoplasty (BT)

BT represents a nonpharmacologic intervention that directly targets the structural component of airway hyperresponsiveness.^337^ The procedure delivers controlled radiofrequency energy via a bronchoscope to the airway wall, reducing airway smooth muscle (ASM) mass and modulating its contractile activity.^338^ Clinical trials, including AIR, RISA, and AIR2, have shown that BT decreases exacerbation frequency, improves asthma-related quality of life, and provides durable benefits for up to ten years of follow-up in selected patients with severe, poorly controlled disease.^339–341^ Mechanistically, BT not only reduces ASM hypertrophy but may also influence local neurogenic and inflammatory signaling, potentially resetting the airway microenvironment.^342,343^ The therapy is indicated for adults with severe asthma inadequately controlled despite maximal pharmacologic treatment and remains the only FDA-approved device-based therapy for asthma.^344^

BT should be performed in carefully selected patients under strict adherence to established indications, with comprehensive assessment of the benefit–risk profile and meticulous peri-procedural safety management. Implementation within clinical research programs or national registries is recommended to ensure optimal outcomes and standardized long-term follow-up.

#### Management of exacerbations

Acute asthma exacerbations, most often triggered by viral infections or allergen exposure, are managed with rapid-acting bronchodilators such as inhaled SABA or nebulized albuterol, sometimes combined with ipratropium. Short courses of systemic corticosteroids are added to quickly reduce airway inflammation and restore control. In severe cases requiring emergency care, additional interventions may include supplemental oxygen, intravenous magnesium sulfate to promote bronchodilation through calcium antagonism, or high-dose β_2_-agonist infusions for refractory bronchospasm. Noninvasive ventilation or intubation becomes necessary when respiratory failure is imminent. Biologic agents are not typically used in the acute setting, although ongoing biologic therapy may be continued if the patient is already receiving it.

#### Addressing resistance

When patients appear unresponsive to standard therapy, the first step is to ensure correct inhaler technique and consistent adherence. Real-world data from smart inhaler studies show that adherence is often <50%, yet digital monitoring with feedback can significantly improve both adherence and asthma control.^345^ It is equally important to confirm that the diagnosis is accurate, since conditions such as vocal cord dysfunction or COPD can mimic asthma and lead to apparent treatment failure. Comorbidities such as chronic sinusitis or gastroesophageal reflux should also be identified and managed, as they frequently worsen symptoms. Only after these basic factors have been optimized should escalation to advanced options such as biologic therapy be considered.

### Biologics and targeted therapies

The era of biologic therapy in asthma began with the recognition of IgE and eosinophils as key drivers in subsets of patients. Biologics are typically monoclonal antibodies designed to neutralize specific cytokines or to block cell surface receptors, thereby interrupting discrete inflammatory pathways (Fig. 9d and Table 3).^346^ They have shown remarkable efficacy in reducing exacerbations and steroid requirements and improving lung function and quality of life in patients with severe T2-high asthma.^20,347^

**Table 3:** Currently available biologics for asthma

	Omalizumab	Mepolizumab	Reslizumab	Benralizumab	Dupilumab	Tezepelumab
Trade name	Xolair	Nucala	Cinqair	Fasenra	Dupixent	Tezspire
Targets	IgE	IL-5	IL-5	IL-5Rα	IL-4Rα	TSLP
FDA	2003	2015	2016	2017	2018	2021
EMA	2005	2015	2016	2018	2017	2022
NMPA	2017	2024	—	2024	2023	2026
Related cell	T_FH_ cell, B-cell	T_H_2 cell, eosinophil	T_H_2 cell, eosinophil	T_H_2 cell, eosinophil	T_H_2 cell	Epithelial cell
Molecular mechanism	Blocks IgE-mediated immune stimulation	Prevents binding of IL-5 to IL-5Rα	Prevents binding of IL-5 to IL-5Rα	Blockade of IL-5Rα ADCC-induced eosinophil apoptosis	Dual receptor antagonism of IL-4/IL-13	Prevents TSLP binding to its receptor complex
Approved ages	≥6 years old	≥6 years old	≥18 years old	≥6 years old	≥6 years old	≥12 years old
Indication	Aeroallergen sensitivity was demonstrated & total IgE of 30-1300 IU/mL	Peripheral eosinophil counts ≥ 150 cells/mL	Peripheral eosinophil counts ≥ 400 cells/mL	Peripheral eosinophil counts ≥ 150 cells/mL	Eosinophilic phenotype OCS-dependent asthma	Severe asthma that is uncontrolled on current therapy
Dosing in adults	dose per IgE & body weight, every 2 to 4 weeks	100 mg, every 4 weeks	3 mg/kg, every 4 weeks	30 mg every 4 weeks for the first 3 doses, then every 8 weeks thereafter	200 mg or 300 mg every 2 weeks; with an initial loading dose depending on regimen	210 mg, every 4 weeks
Route	Subcutaneous	Subcutaneous	Intravenous	Subcutaneous	Subcutaneous	Subcutaneous
Additional indications	CRSwNP; IgE-mediated food allergy; chronic spontaneous urticaria	EGPA; CRSwNP; hypereosinophilic syndrome; COPD with eosinophilic phenotype	—	EGPA	Atopic dermatitis; CRSwNP; eosinophilic esophagitis; prurigo nodularis	—
Key trials	INNOVATE (2004),^431^ EXTRA (2011)^432^	DREAM (2012),^356^ MENSA (2014),^287^ SIRUS (2014),^328^ COMET (2022)^433^	phase Ⅲ trials (2015);^434^ (2016)^435,436^	SIROCCO (2016),^358^ CALIMA (2016),^357^ ZONDA (2017)^329^	LIBERTY ASTHMA QUEST (2018),^365^ VENTURE (2018)^331^	PATHWAY (2017),^21^ NAVIGATOR (2021),^376^ SOURCE (2022)^437^
Efficacy	IgE ↓ Exacerbation ↓ FEV_1_ ↑ QoL and symptom control ↑	Exacerbation ↓ FEV_1_ ↑ Blood and sputum eos ↓ QoL and symptom control ↑ OCS intake ↓	Exacerbation ↓ FEV_1_ ↑ Blood eos ↓ QoL and symptom control ↑ OCS intake ↓	Exacerbation ↓ FEV_1_ ↑ Blood and sputum eos ↓ QoL and symptom control ↑ OCS intake ↓	Exacerbation ↓ FEV_1_ ↑ OCS intake ↓ FeNO ↓	IgE ↓ Exacerbation ↓ Blood eos ↓ FeNO ↓
Limitations	Requires IgE window; SC variability	Less effective if eos < 150	IV only; anaphylaxis risk higher	Less benefit if EOS very low	Transient eosinophilia; conjunctivitis	Cost; long-term real-world data still accumulating

*CRSwNP* chronic rhinosinusitis with nasal polyps, *EGPA* eosinophilic granulomatosis with polyangiitis, *FEV*_1_ forced expiratory volume in 1 s, *QoL* quality of life

#### Anti-IgE (Omalizumab)

Omalizumab is a humanized monoclonal antibody that binds to the FcεRI-binding domain of free IgE, preventing IgE from attaching to mast cells and basophils.^348,349^ It thereby blunts allergic cascade activation. It is indicated for moderate-to-severe allergic asthma with documented perennial allergen sensitivity and elevated IgE levels that are not adequately controlled on ICS. Numerous clinical trials and real-world studies have shown that omalizumab reduces exacerbation rates, emergency visits, and steroid requirements in allergic asthma.^350,351^ It is also beneficial for comorbid allergic rhinitis and has been used in pediatric patients from age 6 up.^352^

In more severe cases, omalizumab has enabled many patients to taper off oral steroids, particularly before the era of newer biologic agents. It is administered subcutaneously every 2–4 weeks at doses based on body weight and pretreatment total IgE levels. A pivotal study in 2003 (INNOVATE) showed an approximately 50% reduction in exacerbations with omalizumab compared to placebo.^353^ The treatment is generally considered when total IgE levels range from 30 to 700 IU/mL, although some guidelines extend this limit to 1500 IU/mL,^354^ and when patients have positive skin or blood tests confirming allergen sensitivity.

Omalizumab targets the extrinsic allergic phenotype, effectively reducing mast cell degranulation and subsequent airway inflammation. By lowering IgE concentrations, it also gradually decreases the number of high-affinity IgE receptors on immune cells over time. The drug has an excellent safety record, with anaphylaxis occurring in fewer than 0.2% of cases, which is why initial doses are given in healthcare settings. With nearly two decades of clinical use, omalizumab has built a strong reputation for safety and efficacy, paving the way for the broader acceptance of biologic therapies in asthma management.

#### Anti-IL-5/anti-IL-5R (eosinophil-targeted therapies)

IL-5 is the key cytokine responsible for eosinophil growth, activation, and survival.^355^ Blocking IL-5 or its receptor has proven to be highly effective in treating eosinophilic asthma.

Mepolizumab is an IgG1 monoclonal antibody that targets IL-5, preventing it from binding to eosinophils and leading to a marked reduction in blood and tissue eosinophil counts. Clinical trials such as DREAM, MENSA, and SIRIUS in patients with severe eosinophilic asthma,^287,328,356^ typically with baseline eosinophil counts ≥ 150 or 300 cells per microliter, demonstrated an approximately 50% reduction in exacerbation rates, along with improvements in quality of life and modest increases in FEV_1_. The SIRIUS trial specifically showed that mepolizumab allowed patients to reduce their maintenance OCS dose by a median of 50%, compared to no reduction in the placebo group.^328^ Mepolizumab is administered subcutaneously at a dose of 100 mg every 4 weeks and is now approved for use in patients aged 6 years and older with severe eosinophilic asthma, often defined by eosinophil counts of at least 150 cells/μL at baseline or 300 within the past year. It has also shown benefits in related eosinophilic disorders such as eosinophilic granulomatosis with polyangiitis and hypereosinophilic syndrome, reflecting its strong suppression of eosinophil activity.

Reslizumab is another monoclonal antibody targeting IL-5, belonging to the IgG4 subclass, and is administered intravenously at a dose of 3 mg/kg every month. Similar exacerbation reductions and lung function improvements were observed in patients with eosinophils ≥400 cells/µL in trials. However, because reslizumab requires intravenous infusion and weight-based dosing, it is used less frequently than mepolizumab or benralizumab.

Benralizumab, an IgG1 monoclonal antibody, acts against the IL-5 receptor α (IL-5Rα) found on eosinophils and basophils. Importantly, benralizumab is afucosylated, enhancing its affinity for FcγRIII receptors on natural killer cells, which triggers antibody-dependent cell-mediated cytolysis (ADCC) of eosinophils and basophils. This unique mechanism leads to near-complete depletion of blood eosinophils to below 50 cells per microliter within days, with similar effects observed in sputum and airway tissues after repeated doses. Clinical studies such as CALIMA,^357^ SIROCCO,^358^ and ZONDA^329^ have shown a 50–70% reduction in exacerbations among patients with severe eosinophilic asthma, along with substantial reductions in OCS use. In the ZONDA study, patients receiving benralizumab achieved a median 75% reduction in OCS dose compared to 25% with placebo, and some patients were even able to discontinue oral steroids entirely.^329^ Benralizumab is administered subcutaneously at a dose of 30 mg every 8 weeks, following two initial doses given 4 weeks apart. Its antibody-dependent cytolytic mechanism may offer advantages such as faster onset of action and possible effects on basophils, which also express the IL-5 receptor.^359^

The clinical response to IL-5 or IL-5 receptor inhibitors is best predicted by blood eosinophil count and the frequency of prior exacerbations. Patients with very high eosinophil levels of 500 cells per microliter or more, along with frequent exacerbations, tend to experience the greatest benefit. Even those with moderate eosinophilia between 100 and 300 cells may show improvement, although to a lesser degree, especially if they are prone to exacerbations.^360^ The presence of nasal polyps is another predictor of good response, as it reflects eosinophil-driven inflammation.

#### Anti-IL-4Rα (Dupilumab)

Anti-IL-4Rα therapy with dupilumab targets both IL-4 and IL-13, which share the IL-4Rα receptor subunit. Dupilumab is an IgG4 monoclonal antibody that blocks this shared receptor, thereby inhibiting signaling from both cytokines.^361^ This broad mechanism interrupts multiple key aspects of type 2 inflammation: it lowers IgE production because IL-4 is needed for class switching, reduces eosinophil recruitment since IL-13 induces eotaxin and both IL-4 and IL-13 indirectly stimulate IL-5 release from ILC2s, decreases mucus production through IL-13’s action on goblet cells,^362^ and lessens bronchial hyperreactivity driven by IL-13.^363,364^

Clinical studies such as LIBERTY ASTHMA QUEST,^365^ which focused on moderate-to-severe asthma, and VENTURE,^331^ which involved oral steroid-dependent asthma, demonstrated that dupilumab significantly reduced exacerbations by approximately 47% overall and by up to 70–80% in patients with high FeNO levels. It also improved lung function by approximately 150–200 mL. In the OCS-dependent population, dupilumab enabled reduction or elimination of oral steroids in a large fraction of patients while also reducing exacerbations.^366^

One notable feature of dupilumab is its effectiveness in both allergic and nonallergic eosinophilic asthma, since IL-4 and IL-13 drive type 2 inflammation regardless of atopic status.^367^ It can even provide benefit in certain cases of so-called “neutrophilic” asthma when IL-13 activity is evident, such as in patients with elevated FeNO, suggesting that some individuals labeled “T2-low” still exhibit airway IL-13 activity, possibly from ILC2 sources.^283,368^ Dupilumab is administered subcutaneously at a dose of 300 mg every two weeks or 200 mg every two weeks in select cases. It is approved for patients aged 12 years and older with moderate-to-severe asthma characterized by an eosinophilic phenotype or dependence on OCS.

Beyond asthma, dupilumab is also used to treat atopic dermatitis and nasal polyps, both of which frequently coexist with asthma.^369^ This dual utility allows it to address multiple type 2 inflammatory conditions simultaneously, supporting the concept of a “unified airway” in which upper and lower airway diseases share common inflammatory mechanisms.^369^ Many patients who present with the triad of asthma, nasal polyps, and aspirin sensitivity have shown dramatic improvement on dupilumab, with both better lung function and noticeable reduction in polyp size, highlighting the central role of IL-4 and IL-13 in these overlapping disorders.^370,371^

#### Anti-IL-13 (Lebrikizumab, Tralokinumab)

Before dupilumab, IL-13–specific antibodies were tried. Lebrikizumab showed FEV_1_ improvement in Phase II, especially in patients with high periostin levels, a marker of IL-13 activity.^372^ However, subsequent phase III trials failed to meet primary exacerbation endpoints, and the development for asthma was stopped.^373^ Tralokinumab, another anti-IL-13, had similar mixed results.^374^ It is thought that blocking IL-13 alone is insufficient, as IL-4 activity continues to be unchecked. Now that dupilumab covers both IL-4 and IL-13, it has replaced these agents. Interestingly, tralokinumab was later found use in atopic dermatitis, where IL-13 plays a major role. Currently, no IL-13-only biologic is approved for asthma, likely because dupilumab’s broader action is more efficacious.

#### Anti-TSLP (tezepelumab)

Tezepelumab is a first-in-class antibody against TSLP, the epithelial alarmin that sits at the top of the inflammatory cascade. TSLP is released by airway epithelial cells in response to various insults and activates dendritic cells, mast cells, basophils, and ILC2s, thereby initiating both allergic and nonallergic inflammation.^375^ Blocking TSLP produces a broad suppressive effect across multiple downstream inflammatory pathways.

In the pivotal NAVIGATOR trial published in 2021,^376^ tezepelumab reduced exacerbations by approximately 56% overall, including in patients with low eosinophil counts where other biologics typically show little benefit.^377^ The reduction reached approximately 70% in high-eosinophil patients and approximately 41% even among those with fewer than 300 eosinophils and low FeNO, representing the T2-low group. This wide-ranging efficacy led to FDA approval for severe asthma regardless of phenotype, making it the first “pan-endotypic” biologic not restricted to eosinophilic or allergic asthma. Patients receiving tezepelumab also showed improvements in lung function and asthma control, and imaging analyses revealed a marked reduction in mucus plug formation on CT scans.^166^

Tezepelumab is administered as a 210 mg subcutaneous injection every four weeks. Real-world data are coming out, but given it’s new, usage is ramping up, particularly for patients who are not obvious candidates for other biologics, such as those with non-eosinophilic but uncontrolled asthma.^378^ It is also a promising choice for individuals with mixed allergic and nonallergic triggers, since TSLP blockade acts upstream and targets inflammation regardless of the initiating factor—be it allergen, virus, or pollutant. The safety profile is favorable, with injection site reactions being the most common side effect. Although TSLP plays a role in host defense, clinical trials have not shown a significant increase in infection risk, aside from a slight, nonsignificant rise in viral respiratory infections. The approval of tezepelumab represents a major step toward endotype-agnostic therapy and offers an important new option for patients with non-T2 asthma, a group that previously lacked effective targeted treatments.

Asthma research is exploring blockade of additional epithelial and inflammatory pathways, particularly for T2-low endotypes.

#### Anti-IL-33 and anti-ST2 (IL-33R)

IL-33 is another epithelial alarmin that powerfully stimulates Th2 and ILC2 responses. Clinical trials have evaluated both anti-IL-33 antibodies, such as itepekimab,^379^ and anti-ST2 antibodies, such as astegolimab,^380^ which block the IL-33 receptor. In a phase II study involving patients with moderate-to-severe asthma, IL-33 inhibition with itepekimab improved lung function and modestly reduced exacerbations compared with placebo.^379^ Astegolimab (anti-ST2), evaluated in the ZENYATTA trial, achieved its primary endpoint by reducing exacerbations in patients with eosinophilic severe asthma.^380^

However, the magnitude of benefit observed with IL-33 pathway blockade has been less pronounced than that seen with IL-5 or IL-4R inhibitors. Ongoing research aims to identify subgroups that may derive the most benefit—potentially those with strong IL-33 activity signatures or with comorbid nasal polyposis. Because viral infections are known to trigger IL-33 release, targeting this pathway may also prove useful in preventing or reducing virus-induced asthma exacerbations.^381^

#### TSLP/IL-33 dual blockade

Given that TSLP, IL-33, and IL-25 often act synergistically, there has been interest in drugs capable of blocking multiple pathways simultaneously. One approach involves bispecific antibodies (e.g., targeting TSLP and IL-13^382–384^ or IL-33 and IL-4Rα^385^). These agents are currently in the preclinical or early trial stages.^386^

#### Anti-IL-17/IL-23

Anti-IL-17 and anti-IL-23 therapies have also been explored in asthma, but the results thus far have been disappointing. Clinical trials targeting IL-17A with secukinumab and IL-17RA with brodalumab in patients with moderate-to-severe asthma failed to show meaningful benefit.^181,387^ Similarly, the IL-23 inhibitor risankizumab did not improve asthma control in a phase II trial.^388^

These findings suggest that although Th17 cells and neutrophils are indeed involved in severe asthma, blocking their key cytokines alone does not lead to significant clinical improvement. This may be due to redundancy in the pathways responsible for neutrophil recruitment or to the masking effects of concurrent corticosteroid therapy. Another explanation could be that these biologics, while effective in Th17-driven diseases such as psoriasis, may not reach lung tissues efficiently or that neutrophilic inflammation in asthma is driven by multiple overlapping signals such as IL-8 and GM-CSF, which are not addressed by IL-17 blockade alone. At present, no biologic therapy has been approved specifically for neutrophilic asthma.

#### Anti-TNF

Because TNFα levels were found to be elevated in certain forms of severe asthma—particularly in patients with systemic inflammation or weight loss suggestive of an autoimmune-like subtype—several trials of anti-TNF therapies such as etanercept and golimumab were conducted between 2006 and 2011.^201,202,389^ These studies, however, failed to demonstrate significant clinical benefit, and some raised safety concerns, including increased risks of serious infections and possible malignancy signals. As a result, anti-TNF therapy is no longer being pursued as a treatment option for asthma.

#### Anti-GM-CSF

Granulocyte-macrophage colony-stimulating factor (GM-CSF) is elevated in the sputum of some severe asthmatics, and it prolongs eosinophil survival and activates alveolar macrophages. An anti-GM-CSF antibody (lenzilumab) was tried in eosinophilic asthma with some benefit in lung function, but development is not far along for asthma specifically.^390,391^

#### Anti-IL-6

IL-6 is a cytokine associated with obesity-related asthma and neutrophilia.^304^ Targeting IL-6 or its receptor is effective in rheumatoid arthritis; in asthma, a trial of tocilizumab (anti-IL-6R) in obese asthmatics is ongoing, given the correlation of IL-6 with neutrophils and corticosteroid insensitivity in that group.^392^

#### TSLP downstream mediators

TSLP activates OX40L on dendritic cells.^178^ A trial of an OX40 antagonist (rocatinlimab) in asthma is underway, aiming to disrupt T-cell costimulation in allergic inflammation.

### Emerging molecular and small-molecule therapies

In parallel with biologics, small-molecule drugs that can be taken orally or inhaled to target specific intracellular pathways are being studied.^393^ Several new therapeutic agents described in the preceding mechanistic sections have already been linked to their signaling or immunologic pathways; accordingly, those agents are not repeated here, and this section focuses on emerging small-molecule and inhaled therapies currently under clinical evaluation.

#### CRTH2 antagonists

CRTH2, also called DP2, serves as the receptor for PGD_2_ on Th2 cells, eosinophils, basophils, and ILC2s. Fevipiprant, an oral CRTH2 antagonist, was considered a promising candidate; early studies demonstrated improved lung function and reduced sputum eosinophils.^394^ However, two large phase III trials, LUSTER 1 & 2, failed to meet their primary endpoints, as the reduction in exacerbations was not significant.^193,395^ The failure may have resulted from issues with dosing, patient selection, since some subjects receiving biologics in those trials confounded the results, or insufficient efficacy of the compound itself.^394–396^ Other CRTH2 antagonists, such as setipiprant, are not currently under active development for asthma. This outcome was disappointing, as CRTH2 blockade could, in principle, address allergic and possibly certain nonallergic type 2 inflammatory pathways.^396^ It underscores the complexity of translating a promising mechanistic target into an effective therapy; redundancy in chemotactic pathways, where alternative chemoattractants may compensate for PGD_2_ blockade, or inappropriate outcome measures can lead to the apparent failure of a pharmacologically active drug.

#### Inhibitors of T-cell trafficking (CCR4, etc.)

Although not prominent in current development pipelines, small molecules or antibodies targeting chemokine receptors expressed on Th2 cells, such as CCR4, which binds TARC/CCL17, have been investigated as potential approaches to reduce Th2 infiltration.^397^ However, none have advanced substantially to date; an anti-CCR4 agent, mogamulizumab, is approved for use in T-cell lymphomas but has not been developed for asthma.^398^

#### Bruton’s tyrosine kinase (BTK) inhibitors

BTK is involved in B-cell receptor and IgE receptor signaling. A phase 2 trial of the oral BTK inhibitor rilzabrutinib in moderate-to-severe uncontrolled asthma showed that although the primary endpoint (loss of asthma control) was not significantly reduced, the drug produced early and sustained improvements in asthma symptoms without increasing infection risk.^399^ This proof-of-concept study suggests that BTK inhibition may represent a novel mechanism for treating uncontrolled asthma, though its risk-benefit balance requires further investigation.

#### SYK inhibitors

Syk kinase is critical for IgE/FcεRI signaling in mast cells.^400^ Fostamatinib, a Syk inhibitor, could theoretically reduce allergen-induced mast cell activation.^401^ However, global immunosuppression and bleeding risk have made systemic Syk inhibitors less appealing for asthma.

#### NF-κB and p38 inhibitors

There were a lot of attempts here. Several p38 MAPK inhibitors (e.g., SCIO-469 and losmapimod) showed lowered inflammation but either liver toxicity or no major clinical effect.^402^ An inhaled p38 inhibitor (AZD7624) in a trial in COPD did not significantly change exacerbations either; again, safety or dose may be issues.^403^ However, the concept remains appealing; a new generation of MAPK inhibitors or targeting MAPK-activated kinases might be explored.^404^

#### Targeting metabolism

There is growing interest in drugs such as metformin, an AMPK activator, for asthma, as retrospective studies have suggested that diabetic patients taking metformin have fewer asthma hospitalizations.^405^ A pilot RCT in obese asthmatics showed some improvement in asthma control and reduced FeNO, possibly by lowering IL-13–induced iNOS, although the results are preliminary. Since metformin is inexpensive and well established, it may gain traction, especially for overweight patients. PPAR-γ agonists such as pioglitazone can also exert anti-inflammatory effects by increasing adiponectin and related pathways. However, one trial in moderate asthma with pioglitazone did not show improvement,^406^ although it might be more beneficial in obesity-related asthma.

GLP-1 receptor agonists are also emerging as a metabolic strategy relevant to asthma, particularly in obesity-related or T2-low phenotypes.^407^ Experimental models show that GLP-1 signaling can attenuate airway inflammation and modulate epithelial alarmin pathways,^408^ while observational clinical studies report improvements in asthma control and fewer exacerbations among obese patients receiving GLP-1 agonists compared with other antidiabetic therapies.^409–411^ Whether these benefits reflect weight loss or weight-independent immunometabolic effects remains under investigation,^407^ but GLP-1 agonists are likely to become increasingly relevant as their respiratory benefits continue to be clarified.

#### Microbiome modulators

Although not small molecules per se, approaches such as probiotics or even bacterial lysate immunotherapies are being explored to reduce asthma exacerbations by training the immune system to become more tolerant and less hyperreactive, somewhat like a “vaccine” against wheezing.^412^ Some pediatric trials of the oral bacterial lysate OM-85 have shown reduced wheezing episodes in toddlers.^413^ However, for established asthma, such strategies remain supplementary at best for now.^414^

### Cellular and gene-based therapies

While pharmacological treatments dominate asthma care, researchers are exploring cell- and gene-based approaches aiming for more durable or curative outcomes by reprogramming the immune system. These are mostly in experimental or early clinical stages.

#### Engineered cell therapies

Engineered immune cell therapy is emerging as a precise strategy to achieve long-term control of airway inflammation. Chimeric antigen receptor (CAR)-based platforms have been designed either to deplete effector cells or to re-establish immune tolerance. Cytokine-anchored CAR-T (CCAR-T) cells using IL-5 as an anchor selectively target IL-5Rα^+^ eosinophils, producing an ~70% reduction of airway eosinophils and sustained efficacy for weeks to months in mice without cytokine-release toxicity.^415^ Eosinophil-derived CCL6 activates CCR1, forming a self-amplifying loop, and blocking this axis augments benefit.^416^ A long-acting construct, 5T_IF_4, integrates CRISPR‒Cas knockout of BCOR and ZC3H12A to prolong CAR-T durability (“pseudoimmortalization”) while secreting an IL-4 mutein that inhibits IL-4/IL-13 signaling; a single infusion maintained efficacy for nearly one year in T2-high models.^417^ Beyond T cells, CAR-NK programs are beginning to appear in asthma.^418^ NK cells can induce eosinophil apoptosis and have a lower theoretical risk of cytokine storm, offering an “off-the-shelf” avenue now being explored in preclinical design and review work. Safety optimization—suicide switches, logic-gate circuits, and local delivery to constrain tissue residency—remains critical, but CAR-T/CAR-Treg/CAR-NK collectively demonstrate the feasibility of durable endotype-targeted immune reprogramming.^419^

#### Stem cell therapies

Stem cell-based interventions exploit the immunomodulatory and reparative properties of mesenchymal and pluripotent cells to attenuate chronic airway inflammation and remodeling. Mesenchymal stem cells (MSCs) from bone marrow, umbilical cord, or adipose tissue suppress Th2 responses via IL-10 and TGF-β, reduce IgE and eosinophil counts, and promote epithelial repair.^420^ Induced pluripotent stem cells (iPSCs) enable personalized airway cell regeneration but require rigorous tumorigenicity control. Resident basal and adult stem cells also contribute to local repair. In murine models, MSCs alleviate inflammation, mucus hypersecretion, and fibrosis; intratracheal or inhaled delivery improves lung targeting. MSC-derived exosomes, notably those enriched in miR-146a, replicate many anti-inflammatory effects and offer a cell-free alternative.^421^ Early clinical studies of umbilical MSC infusion show reduced exacerbations and modest FEV_1_ improvement, although efficacy varies with source, viability, dose, and endotype. Major challenges include long-term safety validation, standardized manufacturing, and enhanced homing through genetic or surface engineering.

#### Gene and nucleic acid therapies

Gene-based strategies aim to intervene at the root of disease by correcting dysregulated pathways rather than suppressing symptoms. Approaches include silencing pro-inflammatory genes, restoring epithelial barrier integrity, and modulating immune cell function. Small interfering RNA (siRNA) or antisense oligonucleotides targeting *IL4*, *IL5*, or *IL13* dampen Th2-driven inflammation, whereas delivery of *IL10* or *IFNG* can re-establish immune balance; repair of barrier genes such as *filaggrin* may reduce allergen penetration and airway hyperresponsiveness. Importantly, human proof-of-concept exists: the GATA3 DNAzyme (SB010) attenuated both early and late asthmatic responses in an allergen-challenge trial.^422^ On the delivery front, peptide-decorated lipid nanoparticles enable epithelial-specific siRNA delivery with downregulation of TSLP/IL-4/IL-13 and mucus programs in vivo,^423^ and AAV6-mediated siRNA has been used preclinically to suppress MUC5AC and mitigate mucociliary dysfunction—prioritizing epithelial/alarmin and mucus axes for local, reversible intervention.^424^ CRISPR‒Cas editing of regulators (e.g., *STAT6*, *NF-κB* nodes) and *FOXP3* reinforcement in Tregs remain attractive but will require stringent control of off-target effects and localization. Given asthma’s polygenic and exposure-dependent architecture, biomarker-guided endotype selection will be essential as these platforms move forward.

Despite many therapeutic candidates failing to reach the clinic, these experiences should not be viewed as wasted efforts. Negative or inconclusive trials often reflect the complexity of asthma rather than the inadequacy of a target. Each attempt contributes to a better understanding of disease mechanisms and guides refinement of trial design, dosing, and patient selection. With emerging biomarkers and more precise phenotyping, some of these approaches may yet find renewed relevance. The scientific rationale for exploring these pathways remains sound, and continued mechanistic study may still yield future therapies capable of altering the course of asthma.

## Future perspectives and clinical outlook

Asthma care has undergone a remarkable evolution, progressing from bench discoveries to bedside applications and now extending into public health and global implementation. Our understanding of the mechanisms underlying asthma, from genetics and epithelial biology to cytokine signaling and neuroimmune interactions, has led to an expanding range of targeted therapies that are transforming outcomes for many patients.

We began by elucidating how genetic predisposition (e.g., *17q21* locus, IL-33 variants) and environmental exposures (allergens, microbes, pollutants) converge on the airway epithelium and immune system to produce distinct asthma phenotypes.^425^ This knowledge gave rise to the concept of asthma endotypes, particularly the dichotomy of T2-high vs T2-low inflammation.^36^ With that came the realization that a one-size-fits-all treatment is suboptimal; instead, therapies must be tailored to these endotypic differences.

Bench research identified critical molecular targets such as IgE, IL-5, IL-4/13, TSLP, and IL-33. The development of biologics against these targets stands as a triumph of translational medicine. Examples include anti-IL-5 antibodies derived from decades of work on eosinophils and anti-TSLP therapies that emerged from studies of epithelial alarmins.^210,415^ These targeted treatments have brought about striking clinical remission in subsets of patients with severe asthma that were once nearly impossible to control.^426^ People who previously experienced frequent hospitalizations and daily symptoms can now achieve near-complete relief and prevention of exacerbations, with some even able to taper off long-term oral steroids.^415^

However, the bench-to-bedside story does not end with introducing new drugs; it continues as an iterative cycle linking research, clinical care, and real-world experience. Insights gained from clinical use, including both successes and failures, feed back into the laboratory to refine mechanistic understanding. For instance, the limited benefit of IL-17 blockade in trials has redirected attention toward alternative neutrophil pathways,^181^ while the broad efficacy of tezepelumab has emphasized the importance of epithelial–immune crosstalk for further investigation.^376^

The future of asthma management lies in precision medicine, not only through selecting the most suitable biologic but also through the comprehensive individualization of care. This approach involves identifying treatable traits, using multi-omics and artificial intelligence to predict and monitor disease, and empowering patients through education and digital tools. The ultimate goal is not just control but remission and potentially even cure. Restoring immune balance in the airways so that the immune system no longer overreacts to harmless stimuli remains the central aim. Achieving this may require long-term induction of tolerance through immunotherapy or Treg enhancement and reversal of airway remodeling through regenerative or sustained anti-inflammatory strategies.

Finally, looking outward, addressing the root environmental and societal causes of asthma remains essential. Reductions in air pollution through clean air regulations have been directly associated with lower asthma incidence and fewer exacerbations; for instance, declines in traffic emissions in some cities have led to measurable improvements in lung function growth among children.^427^ Climate action to curb extreme weather events and CO_2_ rise could mitigate the trend of worsening pollen seasons and wildfire smoke, thereby preventing some asthma morbidity at the population level. Public health efforts such as smoking cessation, obesity prevention, and improved prenatal care to avoid prematurity and low birth weight, which are established asthma risk factors, provide long-term benefits.^428^ The concept of “healthy homes,” including mold remediation, better ventilation, and air purification, has demonstrated reductions in asthma symptoms among disadvantaged communities and is likely to become an increasingly important component of asthma management, extending beyond pharmacotherapy toward prevention and environmental resilience.^429^

In conclusion, the trajectory of asthma research and care is one of increasing personalization and sophistication. From the discovery of IgE and the introduction of inhaled cromolyn in the 1960s to the emergence of combination inhalers and leukotriene antagonists in the 1990s and now to the era of molecular-targeted therapies and digital health integration, each stage has built on previous scientific advances while addressing earlier limitations. Looking forward, asthma management is expected to be guided by comprehensive molecular and clinical profiling, allowing treatment with specific interventions such as biologics, small molecules, or even gene-based therapies, and continuously refined through advanced monitoring technologies. For some patients, it may become possible to prevent progression to severe disease or even achieve lasting remission. Continued translational research, informed by laboratory discoveries of new cytokine pathways and clinical observations of patient heterogeneity, will drive the next generation of therapies. Progress will depend on close collaboration among clinicians, researchers, data scientists, and public health experts to turn these possibilities into reality. With such collective efforts, the long-held belief that “you can’t cure asthma, only control it” may one day be challenged, as science moves closer to altering the natural history of the disease for future generations.
